# Local socio-structural predictors of COVID-19 incidence in Germany

**DOI:** 10.3389/fpubh.2022.970092

**Published:** 2022-09-29

**Authors:** Alisha I. Qamar, Leonie Gronwald, Nina Timmesfeld, Hans H. Diebner

**Affiliations:** Department of Medical Informatics, Biometry and Epidemiology, Ruhr-Universität Bochum, Bochum, Germany

**Keywords:** COVID-19 incidence, SARS-CoV-2, socio-economic risk factors, social determinants of health, public health policy

## Abstract

Socio-economic conditions and social attitudes are known to represent epidemiological determinants. Credible knowledge on socio-economic driving factors of the COVID-19 epidemic is still incomplete. Based on linear random effects regression, an ecological model is derived to estimate COVID-19 incidence in German rural/urban districts from local socio-economic factors and popularity of political parties in terms of their share of vote. Thereby, records provided by Germany's public health institute (Robert Koch Institute) of weekly notified 7-day incidences per 100,000 inhabitants per district from the outset of the epidemic in 2020 up to December 1, 2021, are used to construct the dependent variable. Local socio-economic conditions including share of votes, retrieved from the Federal Statistical Office of Germany, have been used as potential risk factors. Socio-economic parameters like *per capita* income, proportions of protection seekers and social benefit claimants, and educational level have negligible impact on incidence. To the contrary, incidence significantly increases with population density and we observe a strong association with vote shares. Popularity of the right-wing party Alternative for Germany (AfD) bears a considerable risk of increasing COVID-19 incidence both in terms of predicting the maximum incidences during three epidemic periods (alternatively, cumulative incidences over the periods are used to quantify the dependent variable) and in a time-continuous sense. Thus, districts with high AfD popularity rank on top in the time-average regarding COVID-19 incidence. The impact of the popularity of the Free Democrats (FDP) is markedly intermittent in the course of time showing two pronounced peaks in incidence but also occasional drops. A moderate risk emanates from popularities of the Green Party (GRÜNE) and the Christian Democratic Union (CDU/CSU) compared to the other parties with lowest risk level. In order to effectively combat the COVID-19 epidemic, public health policymakers are well-advised to account for social attitudes and behavioral patterns reflected in local popularities of political parties, which are conceived as proper surrogates for these attitudes. Whilst causal relations between social attitudes and the presence of parties remain obscure, the political landscape in terms of share of votes constitutes at least viable predictive “markers” relevant for public health policy making.

## 1. Introduction

Socio-economic conditions have long been known to constitute epidemiological determinants (1), which is also the case within the context of epidemiology of viral infections (2), including the recent COVID-19 pandemic with a focus on incidence (3–5) or on fatalities (6, 7) as dependent outcome. Socio-economic factors such as income, wealth, and education have been spotted as fundamental causes of a wide range of health outcomes (1). However, within the context of the COVID-19 pandemic, researchers' attentions have additionally been drawn to socio-behavioral aspects and political attitudes as crucial predictors of the pandemic activity (8–11). Recently, within this latter context of COVID-19, migration background has been suggested to constitute an additional risk factor for SARS-CoV-2 infection (5) due to, e.g., social isolation and mistrust of the health system. It is likely that all the influencing variables mentioned are more or less strongly correlated with each other. Here, the term “socio-structural” is used to comprise socioeconomic as well as behavioral and political aspects. Knowledge is still rudimentary regarding the combination of the influencing factors mentioned. We here attempt to determine COVID-19 incidence depending on prevailing social attitudes captured by means of affinity to certain political parties and use the most important socio-economic factors in a multivariable regression model to control for possible correlations.

Previous analyses of socioeconomic determinants of COVID-19 incidences (and fatalities) exist for other countries like the USA (6) or Austria (12), however, the results might not be applicable to Germany. Other studies have been conducted at an early pandemic phase and need to be updated to the recent epidemic activity (3, 4, 11). In references (3, 4), a single aggregated parameter, the so called German Index of Socio-economic Deprivation (GISD), has been used as a predictor for COVID-19 incidence. Interestingly, the latter study revealed the more wealthy, i.e., less social deprivated sub-population as an early driver of the epidemic in Germany up to the so called “first wave,” with a change to the opposite in the subsequent course of the epidemic. An obvious reason can be seen in ski tourism, which attracts more wealthy classes of society and which has been identified bearing an important epidemic driver function during the onset of the pandemic (13). In a seroprevalence study restricted to one German region (5), the focus was on ethnicity and adjustments for possible socio-economic confounders, however, leaving comparisons between regions pending. In the latter study, ethnicity as a significant risk factor has been concluded from an overproportionate seroprevalence of the corresponding subpopulation. Having said that, all socio-structural variables are strongly correlated, therefore, Ruck et al. (6) tried to shrink the set of variables down to the statistically most important subset using the least absolute shrinkage and selection operator (LASSO) regression method, however, with ambiguous meaning.

Apparently, Germany currently faces a prevalence of more or less unspecific gestures of political opposition (10), which resulted in the foundation of protest parties like the “Alternative for Germany” (Alternative für Deutschland, AfD). Recent studies suggest that regions with high popularity of the right-wing AfD exhibit higher COVID-19 incidences when being compared to regions with moderate or low AfD popularity (11). Similarly, a study focusing on the impact of vote shares in Austria (12) identifies correlations between political orientations and COVID-19 infection risk and/or mortality. Recent interview-based surveys confirm that radical opponents of anti-corona measures are over-proportionately attracted by the AfD (8–10). However, another large fraction of these opponents is constituted by people with strong concerns with respect to modern medicine or reject some medical interventions like vaccination completely as, e.g., anthroposophists (8–10). Frequently, this group refers to the self-healing power of humans to express their reservations about medical research achievements. Often, this sub-population has an affinity toward the Green Party (Bündnis90/Die Grünen) or similar parties with an emphasis on environmental and bio-ecological aspects (9).

Since previous quantitative analyses left some questions open with respect to potentially correlated socioeconomic factors, we here focus on a multivariable regression model for COVID-19 incidence and refrain from using a score parameter and instead aim at separately assessing crucial socio-structural parameters. Besides the share of votes, the set of covariates includes unemployment rate, educational level, proportion of refugees, proportion of welfare recipients, income, and population density. Among the available census parameters, the proportion of refugees (called “protection seekers” in the German census database) comes closest to the intended consideration of migration background. Including population density is motivated by the hypothesis that metropolitan areas, e.g., might be more prone to high incidences than sparsely populated rural areas. Thus, our analysis adds substantial insights with respect to existing studies, particularly to Richter et al. (11), due to its updating and rigorous methodical extensions.

## 2. Materials and methods

### 2.1. Data

Publicly available data are used exclusively. Three age-stratified time series at the level of 411 rural/urban German districts (Landkreise, kreisfreie Städte) of the registered COVID-19 7-day-incidence (per 100,000 inhabitants) have been retrieved from the database provided by the Robert Koch-Institute (14). Final retrieval date has been Dec 1, 2021. Thereby, the three age classes (in years) [0–14] (referred to as kids), [15–19] (juveniles), and >19 (adults) are used. Of note, due to unobserved COVID-19 cases, these data do not contain true incidences. For the analysis, three episodes of the epidemic time course are used spanning report weeks [41–60], [61–80], and [81–100], respectively, where the report weeks are counted from the first week of 2020 onward. These episodes enclose so called epidemic waves 2, 3, and 4, respectively. Since the peaks of the waves differ among districts, the maximum incidence within each of the periods is used as outcome to be predicted, whereby the period enters the model as one of the independent variables. In a parallelly performed analysis, the cumulative incidences over each of the three periods (“epidemic waves”) have been used as outcomes to be predicted, following the rational that the bulk of each period could be a better measure for the strengths of the epidemic waves. The age class is a further covariate. For brevity, we use “incidence” to refer to the 7-day-incidence per 100,000 inhabitants in the sequel.

Furthermore, socio-economic and census data have been retrieved from the Regional German Database (Regionaldatenbank Deutschland) operated by Federal and State Government Census Bureaus (Statistische Ämter des Bundes und der Länder) (15). The following data, retrieved at the rural/urban district level, have been used as independent variables:

- share of the vote (percentages) resulting from the European election 2019 available for the following parties: CDU/CSU, SPD, GRÜNE, AfD, LINKE, FDP, and Other Parties (cf. see abbreviations) focussing on eligible voters, voter turnout as well as valid second vote. The general vote statistic is established on a full census (Totalerhebung) and uses official transcripts as well as documents from electoral bodies (secondary statistics). In addition, the voter participation has been included to the set of covariates.
- unemployment rate (percentage) regarding the dependent workforce in 2020. The unemployment rate relates the numbers of registered unemployed people to the workforce (workforce and unemployees) as a quota given in percentage. The unemployment rate is focused on the dependent civil workforce, meaning all employees who are subject to social insurance including trainees (Auszubildende), minor (geringfügig) employees, and officials (Beamte) (excluding soldiers) including unemployed people. The data used is based on secondary statistics and gained through administrative processes by a complete survey (Vollerhebung) of regional employment agencies as well as registered people at the Jobcentre.
- graduates of 2019 within the population (percentages) holding particular degrees of education (w/o graduation, Hauptschulabschluss (lower degree secondary education), Mittelschulabschluss [middle degree secondary education), Hochschulreife (higher education entrance qualification)]: The data is based on a full census (Totalerhebung) due to the duty of disclosure for public as well as private schools.
- proportion of the population with the status of protection seekers in 2019 based on data of the Central register of foreigners (Ausländerzentralregister AZR).
- proportion of the population receiving social assistance benefits in 2020 (Empfänger von Hilfe zum Lebensunterhalt) based on a complete survey (Vollerhebung), as well as secondary statistics since already gathered administrative data, is being prepared.
- *per capita* income of private households in Germany from 1995 to 2019 provided by the task force “national accounts of federal states” (Volkswirtschaftliche Gesamtrechnungen der Länder) on behalf of 16 states' statistical offices, federal statistic office, and registration office, Frankfurt. The data used focuses on the primary income of private households including non-profit organizations per inhabitant measured in Euro in 2019.
- population density given by inhabitants per square kilometer (last database update 2021).
- the federal state to which a given district belongs serves as a further determinant.

Of note, data are provided in a consistent way with a nationwide coverage at the spatial levels of entire Germany, 16 federal states, and 411 rural/urban districts (or counties, “Landkreise” or “kreisfreie Städte” in German, respectively). Data are given in a fragmented way at other levels, e.g., cities or metropolitan areas and could, therefore, not be used. The 411 districts are the statutory COVID-19 reporting units which explains why for the regression analysis in the following these districts have been chosen as the “natural” geopolitical units. Furthermore, we added the population density as crucial correlate which allows for an adjustment of differences in agglomeration.

### 2.2. Statistical methods

Within the framework of an ecological study (16), linear random effects regression modeling (17) is used to predict the maximum incidence calculated at the rural district level within one of three pre-defined epidemic periods, depending on the share of the vote. Of note, causal relations usually remain undetermined in ecological studies, which is why the typical paraphrase “prediction model” for a regression in which an outcome depends on an explanatory variable has to be taken with a grain of salt and preferably interpreted as an association. In a parallel (sensitivity) analysis, the cumulative incidences over the periods are used.

In addition, a linear random effects regression is applied in increments at each point in time (i.e., weekly) during the entire observation time in order to obtain the temporal behavior of the regression parameters. Predictors of the regression, i.e., possible risk factors or correlates, are the socio-economic parameters and covariates listed above. The district index has been supplied as random variable. Statistical modeling has been performed using the “lme4” package of “R statistical programming language” (version 4.1.2) (17, 18). We report the estimates along with their *p*-values derived from *t*-statistics (two-sided). Models are compared using likelihood ratio tests (LRT). Significance level is α = 0.05. A summary table of descriptive statistics is presented along with univariate statistical tests (*t*-test for continuous and chi-squared for categorical variables).

The share of votes of all parties add up to 1. Taken as independent variables in a regression analysis, this entails some degree of multicollinearity. Based on the method “leave one variable out” a sensitivity analysis is performed. The full discussion of this matter will be moved to Supplementary Figure S4.

## 3. Results

The summary of socio-structural, demographic, and geographical characteristics included as independent variables in our regression analysis over all 411 rural/urban districts is compiled in Table 1. Hereby, the districts have been separated into districts with AfD share of vote below the median over all 411 districts and share of vote above the median. Obviously, all districts belonging to one of the five East German federal states have AfD share of votes above median. Some of the differences of the districts with high vs. low AfD share of vote with respect to the characteristics considered are significant in terms of univariate tests. Note, the *p*-values resulting from univariate tests are presented here for explorative reasons only, without adjusting for multiple testing. Consequently, some of the characteristics should be considered to be adjusted for in a regression model of COVID-19 incidence with the AfD share of vote as independent variable. This will be rigorously assessed in the following.

**Table 1:** Summary table of characteristics (first column) of rural districts with AfD share of vote below the median value taken over all 411 German districts (second column) and above median (third column), respectively. For an explanation of the characteristics confer the Methods section.

	**≤Median (*N* = 206)**	**>Median (*N* = 205)**	**Total (*N* = 411)**	***p*** **-Value**
**EAST/WEST**				< 0.001
EAST	0 (0.0%)	76 (37.1%)	76 (18.5%)	
WEST	206 (100.0%)	129 (62.9%)	335 (81.5%)	
**Federal state**				< 0.001
Bayern	66 (32.0%)	30 (14.6%)	96 (23.4%)	
Berlin	7 (3.4%)	5 (2.4%)	12 (2.9%)	
Brandenburg	0 (0.0%)	18 (8.8%)	18 (4.4%)	
Bremen	1 (0.5%)	1 (0.5%)	2 (0.5%)	
BW	16 (7.8%)	28 (13.7%)	44 (10.7%)	
Hamburg	1 (0.5%)	0 (0.0%)	1 (0.2%)	
Hessen	9 (4.4%)	16 (7.8%)	25 (6.1%)	
MV	0 (0.0%)	9 (4.4%)	9 (2.2%)	
Niedersachsen	38 (18.4%)	7 (3.4%)	45 (10.9%)	
NRW	34 (16.5%)	19 (9.3%)	53 (12.9%)	
RP	16 (7.8%)	20 (9.8%)	36 (8.8%)	
SA	0 (0.0%)	14 (6.8%)	14 (3.4%)	
Saarland	3 (1.5%)	3 (1.5%)	6 (1.5%)	
Sachsen	0 (0.0%)	13 (6.3%)	13 (3.2%)	
SH	15 (7.3%)	0 (0.0%)	15 (3.6%)	
Thüringen	0 (0.0%)	22 (10.7%)	22 (5.4%)	
**Unemployment**				< 0.001
Mean (SD)	5.65 (2.33)	6.66 (2.55)	6.15 (2.49)	
Range	2.40–12.80	2.20–16.20	2.20–16.20	
**Protection seekers**				0.541
N-Miss	1	2	3	
Mean (SD)	0.02 (0.01)	0.02 (0.01)	0.02 (0.01)	
Range	0.01–0.13	0.00–0.11	0.00–0.13	
**Soc. Benefit claim**.				0.011
N-Miss	1	2	3	
Mean (SD)	0.00 (0.00)	0.00 (0.00)	0.00 (0.00)	
Range	0.00–0.01	0.00–0.01	0.00–0.01	
***Per capita*** **income**				< 0.001
N-Miss	0	1	1	
Mean (SD)	30199.24 (5212.83)	26438.84 (4553.45)	28328.21 (5239.72)	
Range	19048.00–52783.00	18326.00–47353.00	18326.00–52783.00	
**Higher edu**				0.014
Mean (SD)	0.34 (0.10)	0.32 (0.08)	0.33 (0.09)	
Range	0.00–0.59	0.00–0.64	0.00–0.64	
**Without edu**				< 0.001
Mean (SD)	0.06 (0.02)	0.08 (0.03)	0.07 (0.02)	
Range	0.02–0.13	0.03–0.15	0.02–0.15	
**Middle edu**				0.004
Mean (SD)	0.42 (0.07)	0.45 (0.07)	0.43 (0.07)	
Range	0.22–0.61	0.20–0.65	0.20–0.65	
**Low edu**				0.035
Mean (SD)	0.17 (0.05)	0.16 (0.05)	0.17 (0.05)	
Range	0.08–0.40	0.06–0.32	0.06–0.40	
**Population density**				0.052
Mean (SD)	718.38 (982.44)	544.87 (817.98)	631.84 (907.23)	
Range	40.00–4790.00	36.00–4112.00	36.00–4790.00	

**Figure 1:**
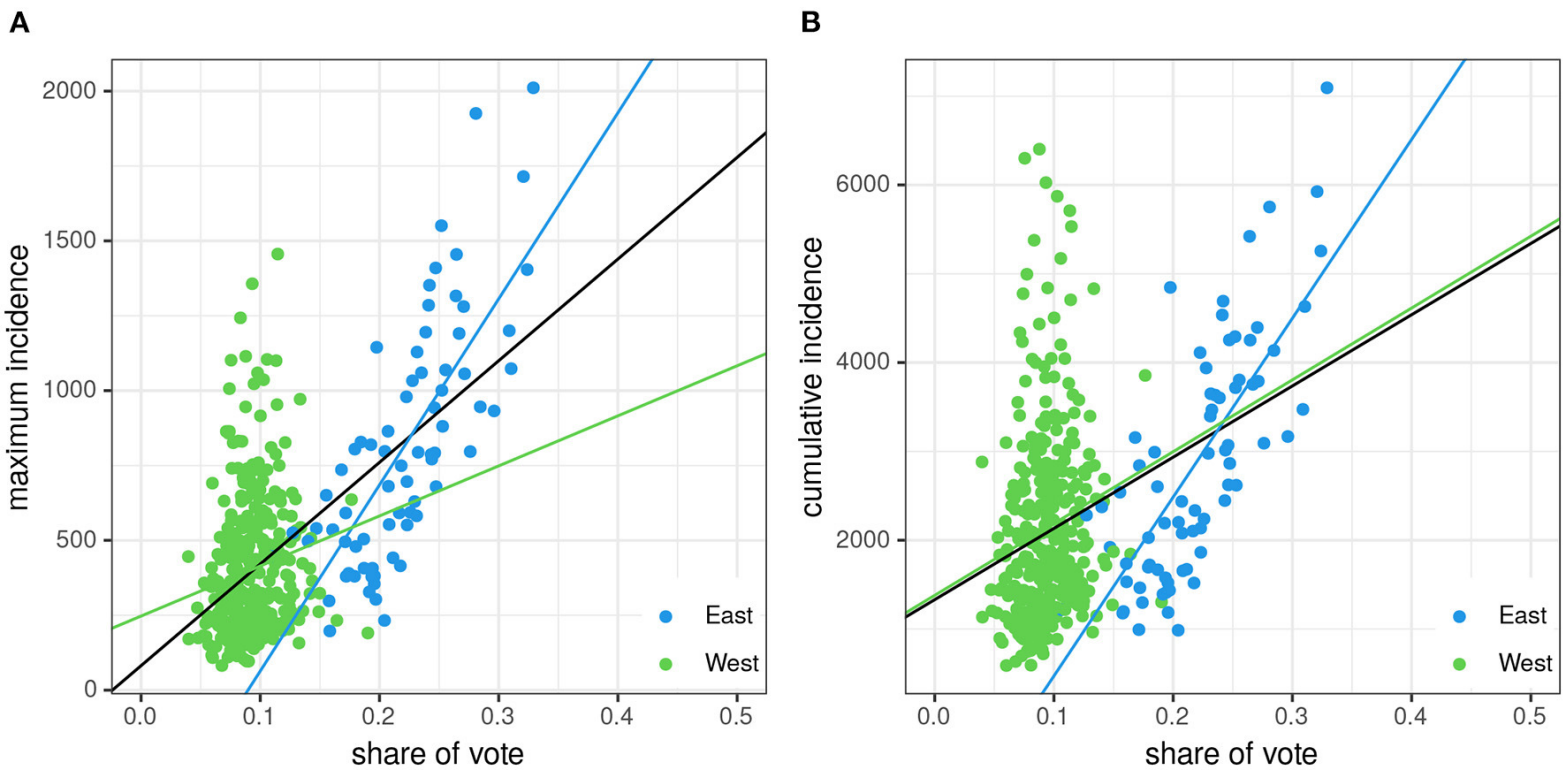
Maximum 7-day-incidence per 100,000 **(A)**/cumulative 7-day-incidence per 100,000 **(B)** within/over the epidemic period [81–100] of the adult population by the AfD share of vote for the 411 rural districts. Data points corresponding to East German districts are depicted in blue, West German districts in green. Three linear regression lines are shown for the full set of points (black), only the East German (blue), and only the West German (green) parts, respectively.

The scatterplot Figure 1A depicts maximum incidence within epidemic period [81–100] of the adult population vs. the share of vote of the AfD for the 411 rural/urban districts. The analogous scatterplot with maximum incidence replaced by cumulative incidence is shown in Figure 1B. The AfD enjoys high popularity in East Germany which gives rise to well-separated point clouds corresponding to these two regions. A comparably strong difference in share of vote between East and West can be observed for the left-wing party “Die Linken” (LINKE), whereas differences in share of vote are more moderate for the other parties, although not negligible (see the full set of scatter plots for the maximum incidence as outcome in Supplementary Figure S1 and cumulative incidence as outcome in Supplementary Figure S2, respectively).

The incidence of a rural/urban district significantly increases with the district's percentage of AfD-vote as shown in Figure 1 by means of linear regression lines. Thereby, we fitted regression lines to the full set of data yielding slopes *s* in Δincidence (*s*_*cum*_ in Δcumulative incidence) per percentage point (*s* = 33.9, *p* < 0.0001; *s*_*cum*_ = 80.19, *p* < 0.0001) as well as to the two subsets belonging to East (*s* = 62.14, *p* < 0.0001; *s*_*cum*_ = 201.3, *p* < 0.0001) and West Germany (*s* = 16.72, *p* = 0.005; *s*_*cum*_ = 80.86, *p* = 0.0027), respectively. It turns out, that the increase of incidence with increasing share of vote is by far stronger in East Germany, which suggests that the regions with their differing socioeconomic conditions might constitute a set of additional, possibly correlated, determinants, which will be analyzed in detail in the following.

In the same vein, for explorative reasons, we performed univariate linear regressions for all the other combinations of the triple set of predictors/correlates: party, epidemic period, age class. Please see the Supplementary Figure S1 for the full set of scatterplots using maximum incidence per period as outcome variable and Supplementary Figure S2 with the cumulative incidence as “bulk” measure of each period as outcome, including regression lines. A qualitative assessment of the results strikingly reveals a dominant impact of the share of vote of the AfD. However, particularly the obvious differences observed for East and West Germany entail a rigorous multivariable regression beyond these explorative univariate analyses, including the required adjustments.

In a first step, a full multivariable linear random effects regression model including the following independent variables is fitted to the age-stratified (using three age classes) COVID-19 incidence data given at rural/urban district resolution:

- share of vote per party excluding “other parties” (cf. section Methods),
- federal state,
- age class with kids as reference,
- epidemic period with weeks [41–60] as reference,
- unemployment rate,
- proportion of protection seekers,
- proportion of social benefit claimants,
- proportion of population with a given level of education excluding the group “w/o graduation,”
- *per capita* income,
- population density.

As before, we parallelly used the maximum incidence and the cumulative incidence as outcome variable, respectively. The resulting parameters and *p*-values are reported using the “/” as separator for the two cases, i.e., “value for maximum/value for cumulative” incidence. Instead of using the two-level factor East/West we switched to the federal state with 16 levels, which proved necessary according to a LRT (*p* < 10^−4^/*p* < 10^−4^). Thus, also North-South gradients in the percentage of votes for distinct parties as well as incidences can be observed. Thereby, unemployment rate, proportion of protection seekers, *per capita* income, as well as the proportion of social benefit claimants all turn out to constitute non-significant risks with *p*>0.55/*p*>0.60 for all corresponding *p*-values obtained by means of a LRT. In addition, skipping all these aforementioned variables simultaneously from the list of covariates and comparing full and reduced model fits yields *p* = 0.98/*p* = 0.93 resulting from a LRT.

Likewise, the educational level does not pose a high risk of elevating the incidence above the average. However, the proportion of the population with a low degree graduation may be conceived as a relevant correlate with an estimate of β = 5.84 (*p* = 0.08)/β = 23.08 (*p* = 0.18), although below statistical significance (see Table 2). Therefore, education is kept within the list of relevant covariates, however, unemployment rate, proportion of protection seekers, and proportion of social benefit claimants are removed due to their irrelevance. To the contrary, population density turns out as a relevant covariate (β = 0.03, *p* = 0.003/β = 0.31, *p* < 0.001), as expected (see Table 2). In full analogy to Supplementary Figures S2, S3 contains all individual univariate correlations “cumulative incidence vs. socio-economic parameter” for all age classes and periods, respectively.

**Table 2:** Result of a linear random effects regression predicting maximum incidence (estimates indicated by “max”) or cumulative incidence (estimates indicated by “cum”), respectively, per epidemic period.

	**Predictor**	**β (max)**	***p*** **-Value (max)**	**β (cum)**	***p*** **-Value (cum)**
Federal state	Bayern	0.395	0.999	3286.174	0.143
	Berlin	-34.611	0.939	3210.232	0.167
	Brandenburg	115.055	0.801	3636.901	0.120
	Bremen	30.711	0.947	3831.774	0.108
	BW	19.867	0.965	3524.750	0.127
	Hamburg	-39.974	0.932	3438.733	0.153
	Hessen	-57.591	0.899	3304.622	0.157
	MV	-63.378	0.890	3019.638	0.201
	Niedersachsen	-43.469	0.924	3118.805	0.183
	NRW	-7.703	0.987	3604.655	0.125
	RP	17.159	0.970	3505.172	0.129
	SA	140.166	0.756	3651.953	0.115
	Saarland	96.533	0.834	3991.444	0.092
	Sachsen	287.240	0.529	4431.647	0.059
	SH	-143.821	0.750	2434.284	0.293
	Thüringen	123.857	0.788	4271.552	0.071
Age class	Juveniles	78.969	<0.001	839.001	<0.001
	Adults	-112.772	<0.001	-239.226	<0.001
Period	[61–80]	-3.253	0.759	-374.344	<0.001
	[81–100]	445.519	<0.001	1217.538	<0.001
Education level	Low edu	5.842	0.080	23.084	0.177
	Middle edu	3.735	0.199	12.294	0.409
	High edu	4.091	0.152	15.196	0.298
	Population density	0.033	0.003	0.312	<0.001
Share of vote	AFD	24.537	<0.001	101.449	<0.001
	SPD	-9.256	0.023	-58.548	0.005
	CDU	2.263	0.552	2.694	0.890
	GRÜNE	3.242	0.480	8.521	0.717
	LINKE	-12.623	0.027	-107.939	<0.001
	FDP	-16.895	0.010	-100.145	0.003
	Vote participation	-4.417	0.002	-39.023	<0.001

Reference levels of the categorical predictors are: kids for age class and weeks (41–60) for epidemic period. The metric covariates are given in %, thus the corresponding βs have to be interpreted as Δincidence (or Δcumulative incidence, respectively) per percentage point. Due to the constraints that the percentages corresponding to share of vote and education, respectively, sum up to 100% we skipped the variables “Other Parties” and “w/o graduation” in order to avoid singularities. The random effect of the rural districts yields a standard deviation of 28.23 (“max”) and 319.7 (“cum”) for the intercept and 262.4 (“max”) and 1036.6 (“cum”) residual.

The reduced model resulting from the model reduction process described above yields the results listed in Table 2. The most striking result of the regression is the highly significant effect of the AfD's share of vote for the prognosis of COVID-19 incidence. Even after inclusion of several socioeconomic and epidemiological covariates, a strong risk of high incidences can be observed when being compared to the share of vote of other parties.

Due to the constraint that the percentages sum up to 100%, the impact has to be interpreted in a relative sense. More specifically, this constraint may entail some degree of multicollinearity [cf. (19)]. However, a perfect multicollinearity would be present if and only if the coefficients of collinearity would be identical for all districts. In contrast, the 411 German rural/urban districts exhibit considerable heterogeneity in terms of popularity of parties expressed by their share of votes. A common procedure to assess the degree and impact of multicollinearity is to drop one of the variables from the set of covariates. The full discussion of the results obtained from a corresponding sensitivity analysis is moved to Supplementary Figure S4. To summarize the result, leaving one party out leads to an approximately constant shift of the values of the regression parameters of the remaining covariates, whereby the magnitude of the observed shift depends on the omitted party. The observation of such a bias in moderately collinear covariates is a known phenomenon, consequently, the estimates have to be interpreted in a relative sense. Inferences drawn from these results are unchanged when being compared with the inferences drawn from the full model.

In this line, CDU/CSU as well as GRÜNE rank between AfD and the other parties in terms of the magnitude of risk. Obviously, rural/urban districts with high percentages of votes of the AfD exhibit characteristics that constitute risk factors for COVID-19 incidence. The increasing risk resulting from an increasing popularity of CDU/CSU and GRÜNE is more moderate or neutral, whereas the increasing percentages of the other parties seem to unfold a lowering in risk of incidence. Independently, of whether the local characteristics that determine higher incidence rates are directly related to the political agenda of the corresponding parties or not, the very fact of increased incidences entails that the politicians are in charge to reflect these characteristics of their districts.

The impacts of most of the other covariates are not very surprising. Saxony (Sachsen) faced the by far highest incidence during the fourth wave (weeks [81–100]), whereas Schleswig-Holstein (SH) exhibited an incidence well below the country average, consistent with the observed statistical significance of the two federal states and the epidemic period, respectively. Of note, an increasing vote participation turns out to statistically significantly lower the risk of COVID-19 incidence. A shared hostile stance with respect to public health and other policies between AfD voters and non-voters might by a possible, although speculative interpretation. To complete the report, the random effect of the rural/urban districts yields a standard deviation of 28.23/319.7 for the intercept (262.4/1036.6 residual), hence pointing to a considerable random variation between the districts not captured by the fixed effects of the model.

**Figure 2:**
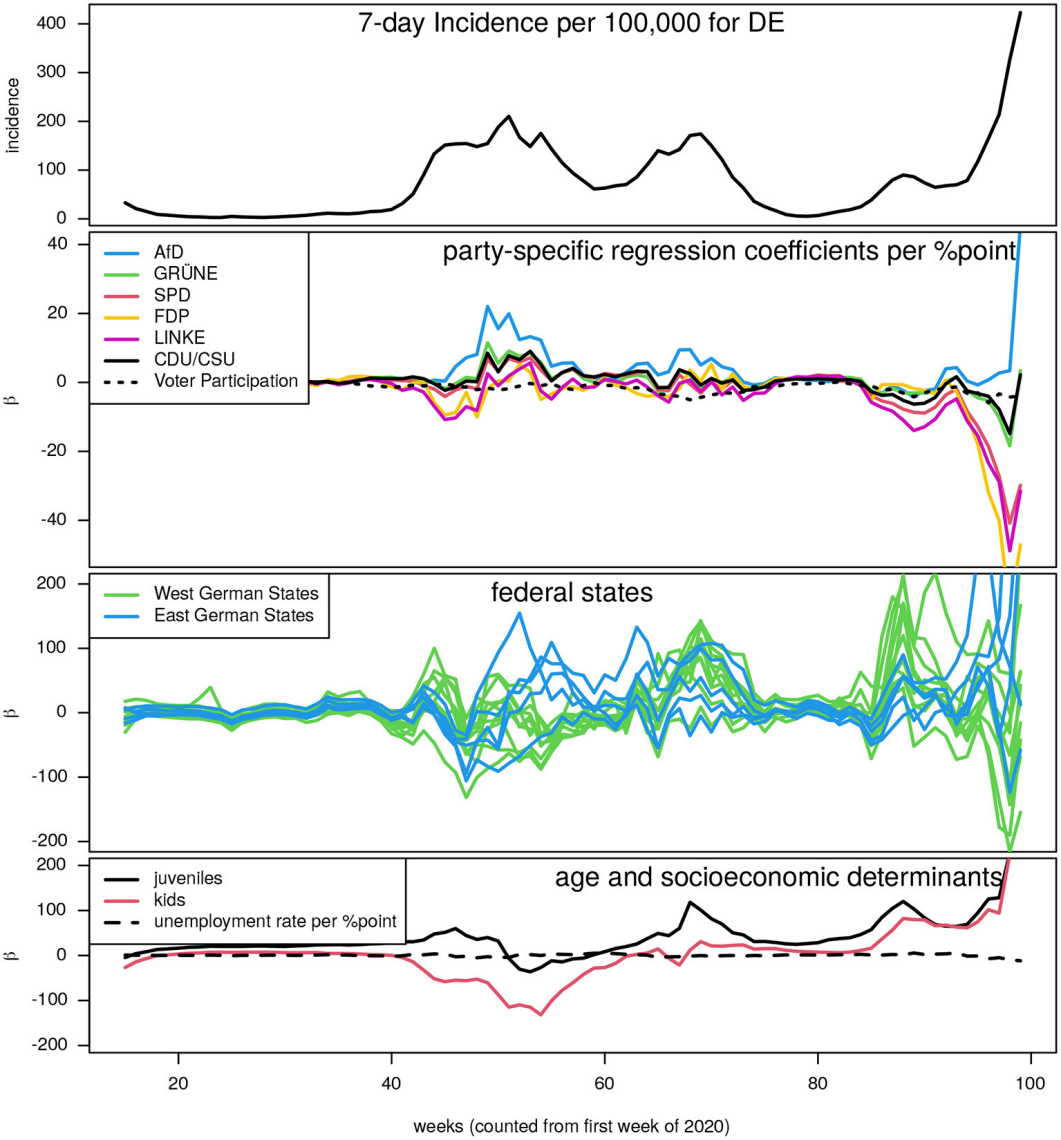
Time courses of regression parameters obtained from random effect linear regression modeling sequentially applied at all available time points during the observation time. Panel on top: the German COVID-19 7-day-incidence per 100,000 curve (to allow for a mapping of the results to the epidemic history). Other panels from top to bottom: regression parameters in units “per percentage point” corresponding to (i) the share of votes (including voter participation), (ii) the federal states (for a better visibility all West German states depicted in green, East German states in blue), (iii) age classes (adults are reference) and percentage of unemployment.

In a second explorative approach, we applied the random effects linear regression at each instant of time in order to obtain time series of the regression parameters. The rational behind doing so is to reveal possible temporal effects with impact on the prediction. The result is shown in Figure 2. The dominant role of the AfD share of vote in being positively correlated with incidences can be confirmed: the magnitude of the corresponding regression parameter almost persistently remains on top from end of 2020 on (Figure 2, second panel). This “time-dependent” regression also reveals a short period (around week 90) in which the Free Democratic Party (FDP) is on top. The moderate risk emanating from the GRÜNE and CDU/CSU can now apparently be attributed to the last episode roughly from week 90 onward.

The remaining two panels of Figure 2 are devoted to show the time courses of estimated effects corresponding to federal state, age classes, and unemployment rate. The latter effect remains well below significance throughout the entire observation time and has here been chosen as a proxy for all the other socio-economic determinants. All included socio-economic parameters do not show any role on predicting COVID-19 incidence after week 40. The federal states as well as the age classes, however, turn out to be significantly predictive from this time-dependent version of regression modeling as well, as already shown above where we aggregated the incidence time series into maximum or cumulative values of three dominating periods. Apparently, phases of higher and lower incidences alternate in an almost reciprocal way between West and East German states. Kids and juveniles play an increasing role as correlate with high incidences, most likely due to the increasing vaccination rate of the adult cohorts, but periods of school closures and openings and unstable interventions may also play a decisive role.

## 4. Discussion

We have shown that a multivariable linear random effects regression modeling yields popularity of the AfD (in terms of share of the vote) as a covariate significantly correlated with high incidence even after adjustment for several socioeconomic covariates. In contrast, an increasing percentage of votes for most of the other parties is associated with a reduced COVID-19 incidence with the exceptions of Christian Democratic Union (CDU/CSU) and the Green Party (GRÜNE) whose percentage of votes are insignificant in absolute terms for predicting the incidence. However, due to the constraint that percentages of votes sum up to 100% the results have to be interpreted in a relative sense. Thus, in relative terms, popularity of the AfD is strongly correlated with an increase in incidence, whereas popularity of CDU/CSU or GRÜNE is associated with medium risk, respectively, and the lowest risk emanates from the other parties.

One of the limitations of this analysis is the fact that correlations do not allow to draw inferences on causality. However, a comprehensive sociological interpretation is beyond our aim which is driven by the demand to supply relevant information for public health policies. From this point of view it is crucial to be able to spot locally given conditions that are informative for epidemiological control strategies, whether these conditions have causal or mere correlative structures.

Furthermore, our analysis is limited by the fact that the officially registered incidences depend on local COVID-19 test policies and the corresponding infrastructural conditions. Policies with respect to opening of schools and corresponding test strategies are particularly important since the impact of kids and juveniles as possible epidemic drivers is controversially debated (20–25). However, it seems more plausible that the average frequency of testing is even less in regions with a high “anti-corona attitude” prevalence, which thus would even amplify our result. Having that said, an in-depth analysis of the impact of both children-related policies and epidemic dynamics is encouraged.

In addition, the usage of aggregated data (proportions and population averages) might limit validity. Therefore, we engage the reader in relating our result with the insights gained by surveys based on individual interviews (8–10). We focused on predicting local incidences, however, it is suggested to also include fatalities in future studies [for a seminal work see (6, 7)].

Another limitation is the neglect of pandemic-relevant working conditions or high-risk occupations. For example, meat processing plants proved to be pandemic hotspots both in Germany and the USA (26–28). As far as Germany is concerned, there is a lack of reliable information on the spatial distribution of corresponding industrial branches. Access to reliable information regarding the spatial coverage of nursing homes and comparable vulnerable facilities turns out to be similarly problematic. However, a strong correlation with the spatial distribution of vote shares does not seem very plausible. More generally, due to limited availability of detailed socio-cultural variables and in order to avoid difficult to analyze hierarchical correlation structures of these variables, we have decided to differentiate the common German index of socio-economic deprivation only to a manageable degree by including the arguably most important components. We further assume that controlling for federal states and the selected socio-structural variables constitutes a sufficiently good basis for a reliable regression. However, we advocate conducting in-depth multi-variable analyses as reliable data becomes available.

Finally, we did not include vaccination coverage in our analysis due to unavailable high-quality data with required spatial resolution. However, rough estimates regularly published by the Robert Koch-Institute on their online COVID-19 dashboard (29) suggest that incidence and vaccine coverage is negatively correlated. In addition, vaccine coverage might have an impact on hospitalization and severe COVID-19 illness, but is arguably considerably less important as protective factor for asymptomatic infections since the SARS-CoV-2 immunization is generally not sterile. Importantly, in our context, AfD-politicians officially propagate an anti-vaccination attitude consistent with findings in related surveys (8–10), which thus renders a significant impact of vaccination coverage on our main result as very unlikely.

In the same line, other locally differing COVID-19 containment strategies may play roles in predicting incidences. However, thorough research to determine locally applied containment measures revealed inconsistency coupled with opaque documentation. In this context, it is appropriate to point out the discrepancy between rule and compliance and it appears to be likewise important to determine which factors drive (non-)compliance (30), so that we have again arrived at the political culture. Once again, it is plausible that most of these local differences are already contained in the popularities of certain parties as appropriate surrogate measures. Generally, the legislative responsibility for containment measures is at the federal state level in Germany. Therefore, as mentioned above within the context of the industrial landscape, we are convinced that the federal states already take sufficient account of the need for adjustment. A reliable evaluation of the aforementioned aspects within the scope of our ecological study appears impracticable, therefore, we refrain from comprehensive sociological analyses and refer to published work instead (8–11, 31) where motivations of the protest movement have been discussed. In reference (31), the Austrian situation is discussed revealing an impact of the right-wing party FPÖ similar to the German AfD. In following the cited literature, the prevalence of conspiratorial attitudes is above average among the new protest movement which might in turn be intensified by the extensive use of new media communication tools [cf. (32, 33) for a critical discourse, also see (31)].

Of note, we refrained from presenting an analog analysis using logarithmized 7-day-incidences as outcome since checks hereof led to irrelevant differences only. Finally, we did not consider spatial correlations. Although we do not regard this as a serious limitation it might be worthwhile to elaborate on this aspect in future studies based on spatial regressions.

## 5. Conclusions

Conclusive inferences have to be drawn with utmost care. The presented analysis of sociostructural risk factors aims in informing public health and epidemiology policymakers. We refrain from any accusation which appears to be inappropriate due to unclear causality. We use the share of the vote of a particular party as an approximate surrogate parameter that presumably captures sociobehavioral aspects and correlates with COVID-19 incidence beyond other socioeconomic factors and we strongly advocate a subsequent reflection of our results from a sociopolitical perspective, including representatives of the corresponding parties. We adopt the conclusions from a similar study (12) focusing on the impact of vote shares in Austria: “While these parameters are apparently only single elements of complex causal chains that finally lead to individual susceptibility and vulnerability levels, our findings might have identified ecological parameters that can be utilized to develop fine-tuned communications and measures in upcoming challenges of this and other pandemics.”

Specifically, locally observed high COVID-19 incidences are associated with local popularity of the right-wing party AfD. Multivariable linear random effects modeling with adjustments for the most important socio-economic public-health determinants and the inclusion of epidemiological covariates yields a high degree of reliability of this result. It is particularly worth of note that a set of the most important socio-economic factors plays a minor role in driving the epidemic. As expected, population density has a statistically significant impact on COVID-19 incidence, however, an adjustment of estimates of the other correlates including the share of votes cannot be observed and we thus conclude that the share of votes are not correlated with population density. Speculatively, social and anti-governmental attitudes play a more important role where the popularity of a party can be conceived as a proper surrogate measure [cf. (8, 10)].

Local popularities of other parties by means of their share of vote lead to much weaker or even negative associations with COVID-19 incidences within the corresponding rural districts, with the exceptions of CDU/CSU and GRÜNE. In addition, during a short period of time the popularity of the FDP appears to pose a risk of increased COVID-19 incidence.

To conclude, COVID-19 incidence appears to be age-dependent. Incidence is higher amongst adolescents when being compared with the younger kids and the adults throughout the course of the epidemic. In agreement with the information provided at the online dashboard operated by the Robert Koch-Institute [RKI, (29)], the adults' incidence continued to stay below the children's incidence from the start of the vaccination campaign onward. However, the age-stratified incidence curves exhibit a waxing and waning in the course of time which certainly reflects corresponding regulations at schools and daycare facilities for children. Partially, age-specific measures and regulations also depend on local policies and, therefore, at least in part on locally prevalent social attitudes. Thus, we herewith encourage further studies into spatio-temporal epidemic dynamics that account for the spatio-temporal variability of related epidemiological containment policies which is urgently needed for a comprehensive understanding not only of the SARS-CoV-2/COVID-19 pandemic but also for being prepared for similar potentially disastrous public health challenges in the future.

## Data availability statement

Publicly available datasets were analyzed in this study. This data can be found here: survstat.rki.de and regionalstatistik.de.

## Author contributions

HD and NT: conceptualization, methodology, formal analysis, and supervision. HD: software and writing–original draft preparation. AQ and LG: resources and data curation. HD and AQ: visualization. NT: project administration. All authors: validation and writing–review and editing. All authors have read and agreed to the published version of the manuscript.

## Funding

The APC was funded by Open Access Publication Funds of the Ruhr-Universität Bochum.

## Conflict of interest

The authors declare that the research was conducted in the absence of any commercial or financial relationships that could be construed as a potential conflict of interest.

## Publisher's note

All claims expressed in this article are solely those of the authors and do not necessarily represent those of their affiliated organizations, or those of the publisher, the editors and the reviewers. Any product that may be evaluated in this article, or claim that may be made by its manufacturer, is not guaranteed or endorsed by the publisher.

## Abbreviations

GISD: German index of socio-economic deprivation
BW: Baden Württemberg
MV: Mecklenburg-Vorpommern
NRW: Nord-Rhein-Westfalen
RP: Rheinland-Pfalz
SA: Sachsen-Anhalt
SH: Schleswig-Holstein
Low edu: lower degree secondary education
Middle edu: middle degree secondary education
High edu: higher education entrance qualification
AfD: Alternative for Germany
SPD: Social Democratic Party of Germany
CDU/CSU: Christian Democratic Union of Germany
FDP: Free Democratic Party
LINKE: The Left Party, Die Linke
GRÜNE: The Green Party, Bündnis 90/Die Grünen.

## References

[1] Braveman P Gottlieb L. The social determinants of health: it's time to consider the causes of the causes. Publ Health Rep. (2014) 129:19–31. 10.1177/00333549141291S206 24385661 PMC3863696

[2] Chandrasekhar R Sloan C Mitchel E Ndi D Alden N Thomas A. Social determinants of influenza hospitalization in the United States. Influen Other Respir Virus. (2017) 11:479–88. 10.1111/irv.12483 28872776 PMC5720587

[3] Wachtler B Hoebel J. Soziale Ungleichheit und COVID-19: sozialepidemiologische Perspektiven auf die Pandemie. Gesundheitswesen. (2020) 82:670–5. 10.1055/a-1226-6708 32858757

[4] Hoebel J Michalski N Wachtler B Diercke M Neuhauser H Wieler LH. Socioeconomic differences in the risk of infection during the second SARS-CoV-2 Wave in Germany. Dtsch Arztebl Int. (2021) 118:269–70. 10.3238/arztebl.m2021.0188 34114548 PMC8287075

[5] Brinkmann F Diebner HH Matenar C Schlegtendal A Eitner L Timmesfeld N. Seroconversion rate and socioeconomic and ethnic risk factors for SARS-CoV-2 infection in children in a population-based cohort. Euro Surveill. (2022) 27:2101028. 10.2807/1560-7917.ES.2022.27.37.2101028 36111557 PMC9479468

[6] Ruck DJ Bentley RA Borycz J. Early warning of vulnerable counties in a pandemic using socio-economic variables. Econ Hum Biol. (2021) 41:100988. 10.1016/j.ehb.2021.100988 33636583 PMC8054145

[7] Morwinsky S Nitsche N Ph. D. Acosta E. COVID-19 fatality in Germany: demographic determinants of variation in case-fatality rates across and within German federal states during the first and second waves. Demogr Res. (2021) 45:1355–72. 10.4054/DemRes.2021.45.45

[8] Nachtwey O Schäfer R Frei N. Politische Soziologie der Corona-Proteste. SocArXiv. (2020) 12:zyp3f. 10.31235/osf.io/zyp3f

[9] Nachtwey O Frei N Markwardt N. “Querdenken”: Die erste wirklich postmoderne Bewegung. Oliver Nachtwey und Nadine Frei, im Interview mit Nils Markwardt. Philosophie Magazin Online (2021). Available online at: https://www.philomag.de/artikel/querdenken-die-erste-wirklich-postmoderne-bewegung (accessed December 20, 2021).

[10] Schäfer R Frei N. Rationalismus und Mystifikation: zur formalen Pathetik des Dagegenseins. Z Religion Ges Polit. (2021) 5:391–410. 10.1007/s41682-021-00095-9 34938949 PMC8620311

[11] Richter C Wächter M Reinecke J Salheiser A Quent M Wjst M. Politische Raumkultur als Verstrker der Corona-Pandemie? Einflussfaktoren auf die regionale Inzidenzentwicklung in Deutschland in der ersten und zweiten Pandemiewelle 2020. Z Rechtsextremismus. (2021) 2021:S.191–S.211. 10.3224/zrex.v1i2.01A

[12] Moshammer H Poteser M Weitensfelder L. COVID-19: regional differences in Austria. Int J Environ Res Public Health. (2022) 19:1644. 10.3390/ijerph19031644 35162665 PMC8835493

[13] Knabl L Mitra T Kimpel J Rssler A Volland A Walser A. High SARS-CoV-2 seroprevalence in children and adults in the Austrian ski resort of Ischgl. Commun Med. (2021) 1:4. 10.1038/s43856-021-00007-1 34870284 PMC8633917

[14] Robert Koch-Institute. SurvStat@RKI 2.0 (2022). Available online at: survstat.rki.de (accessed January 26, 2022).

[15] Regionaldatenbank Deutschland. Statistische Ämter des Bundes und der Länder (2022). Available online at: regionalstatistik.de (accessed January 6, 2022).33596931

[16] Morgenstern H. Ecologic studies in epidemiology: concepts, principles, and methods. Annu Rev Publ Health. (1995) 16:61–81. 10.1146/annurev.pu.16.050195.000425 7639884

[17] Bates D Mächler M Bolker B Walker S. Fitting linear mixed-effects models using lme4. J Stat Softw. (2015) 67:1–48. 10.18637/jss.v067.i01

[18] R Core Team. R: A Language Environment for Statistical Computing. Vienna: R Core Team (2021). Available online at: https://www.R-project.org/

[19] Farrar DE Glauber RR. Multicollinearity in regression analysis: the problem revisited. Rev. Econ Stat. (1967) 49:92–107.

[20] Brinkmann F Diebner HH Matenar C Schlegtendal A Spiecker J Eitner L. Longitudinal rise in seroprevalence of SARS-CoV-2 infections in children in Western Germany–a blind spot in epidemiology? Infect Dis Rep. (2021) 13:957–64. 10.3390/idr13040088 34842714 PMC8629019

[21] Diebner HH. Phase shift between age-specific COVID-19 incidence curves points to a potential epidemic driver function of kids and juveniles in Germany. medRxiv. (2021). 10.1101/2021.11.29.21267004

[22] Berger U Fritz C Kauermann G. Schulschließungen oder Schulöffnung mit Testpflicht? Epidemiologisch-Statistische Aspekte Sprechen für Schulöffnungen mit Verpflichtenden Tests. CODAG Bericht Nr. 14 vom 30.04.2021. München: Uni München (2021). Available online at: www.covid19.statistik.uni-muenchen.de/pdfs/codag_bericht_14.pdf

[23] Klüver H Hartmann F Humphreys M Geissler F Giesecke J. Incentives can spur COVID-19 vaccination uptake. Proc Natl Acad Sci USA. (2021) 118:e2109543118. 10.1073/pnas.2109543118 34413212 PMC8433545

[24] Martinoli C La Vecchia C Raimondi S Bellerba F Sasso C Basso A. SARS-CoV-2 circulation in the school setting: a systematic review and meta-analysis. medRxiv. (2021). 10.1101/2021.09.03.21263088 35564779 PMC9099553

[25] Brailovskaia J Schneider S Margraf J. To vaccinate or not to vaccinate!? Predictors of willingness to receive Covid-19 vaccination in Europe, the U.S., and China. PLoS ONE. (2021) 16:e0260230. 10.1371/journal.pone.0260230 34851986 PMC8635370

[26] Günther T Czech-Sioli M Indenbirken D Robitaille A Tenhaken P Exner M. SARS-CoV-2 outbreak investigation in a German meat processing plant. EMBO Mol Med. (2020) 12:e13296. 10.15252/emmm.202013296 33012091 PMC7646008

[27] Middleton J Reintjes R Lopes H. Meat plants—a new front line in the covid-19 pandemic. BMJ. (2020) 370:m2716. 10.1136/bmj.m2716 32646892

[28] Herstein JJ Degarege A Stover D Austin C Schwedhelm MM Lawler JV. Characteristics of SARS-CoV-2 transmission among meat processing workers in Nebraska, USA, and effectiveness of risk mitigation measures. Emerg Infect Dis. (2021) 27:1032–8. 10.3201/eid2704.204800 33591249 PMC8007314

[29] Robert Koch-Institut. COVID-19-Dashboard (2022). Available online at: https://npgeo-corona-npgeo-de.hub.arcgis.com/ (accessed April 16, 2022).

[30] Six F de Vadder S Glavina M Verhoest K Pepermans K. What drives compliance with COVID-19 measures over time? Explaining changing impacts with goal framing theory. Regul Gov. (2021) 2021:10.1111/rego.12440. 10.1111/rego.12440 34909051 PMC8661714

[31] Brunner M Daniel A Knasmüller F Maile F Schadauer A Stern V. Corona-protest-report. Narrative – Motive – Einstellungen. SocArXiv. (2021). 10.31235/osf.io/25qb3

[32] Diebner HH. Kunstvergessenheit. Oder: die systemwissenschaftliche Vernutzung von Kunst. In:Schläder J Weber F, editors. Gegenwelten - Zwischen Differenz und Reflexion. Leipzig: Henschel-Verlag (2009), 84–121.

[33] Diebner HH. Digital technology as matrix for constructivism and verdinglichung. Studia UBB Philos. (2010). LV/3:33–59.

