# Supplementary material for: Local socio-structural predictors of COVID-19 incidence in Germany
Source: Front Public Health. 2022 Sep 29;10:970092. doi: 10.3389/fpubh.2022.970092 (PMC9556738; doi:10.3389/fpubh.2022.970092)

Supplement S1 to:

Alisha I. Qamar, Leonie Gronwald, Nina Timmesfeld, and Hans H. Diebner: Local Sociostructural Predictors of COVID-19 Incidence in Germany

The following series of figures shows age-stratified (3 age classes, *age*) locally observed **maximum** incidences of 3 pre-defined epidemic periods (*p*) versus the share of vote (2019 European Parliament election) of 7 political parties and voter participation (*party*), respectively. Specifically, each panel depicts a scatterplot of maximum incidence for a pair [*p*, *age*] by share of vote of a selected *party* for 411 German rural districts. Data points corresponding to East German districts are depicted in blue, West German districts in green. Three linear regression lines are shown for the full set of points (black), only the East German (blue), and only the West German (green) parts, respectively.
The header of each page contains the given pair [*p*, *age*], 9 in total, and each panel is labeled by the name of the selected *party*.

Max. Incidence (kids) vs share of vote for period [41-60]

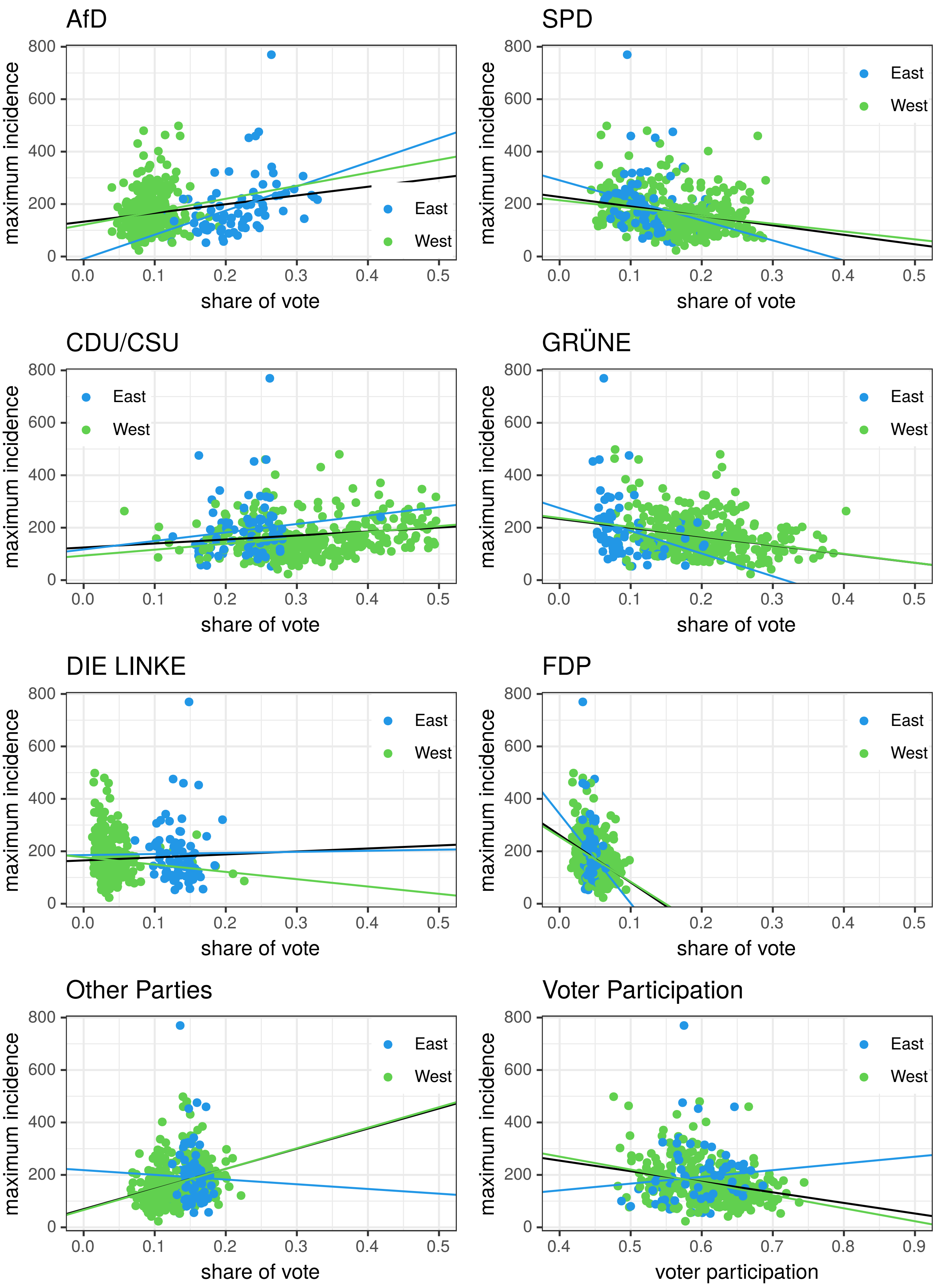

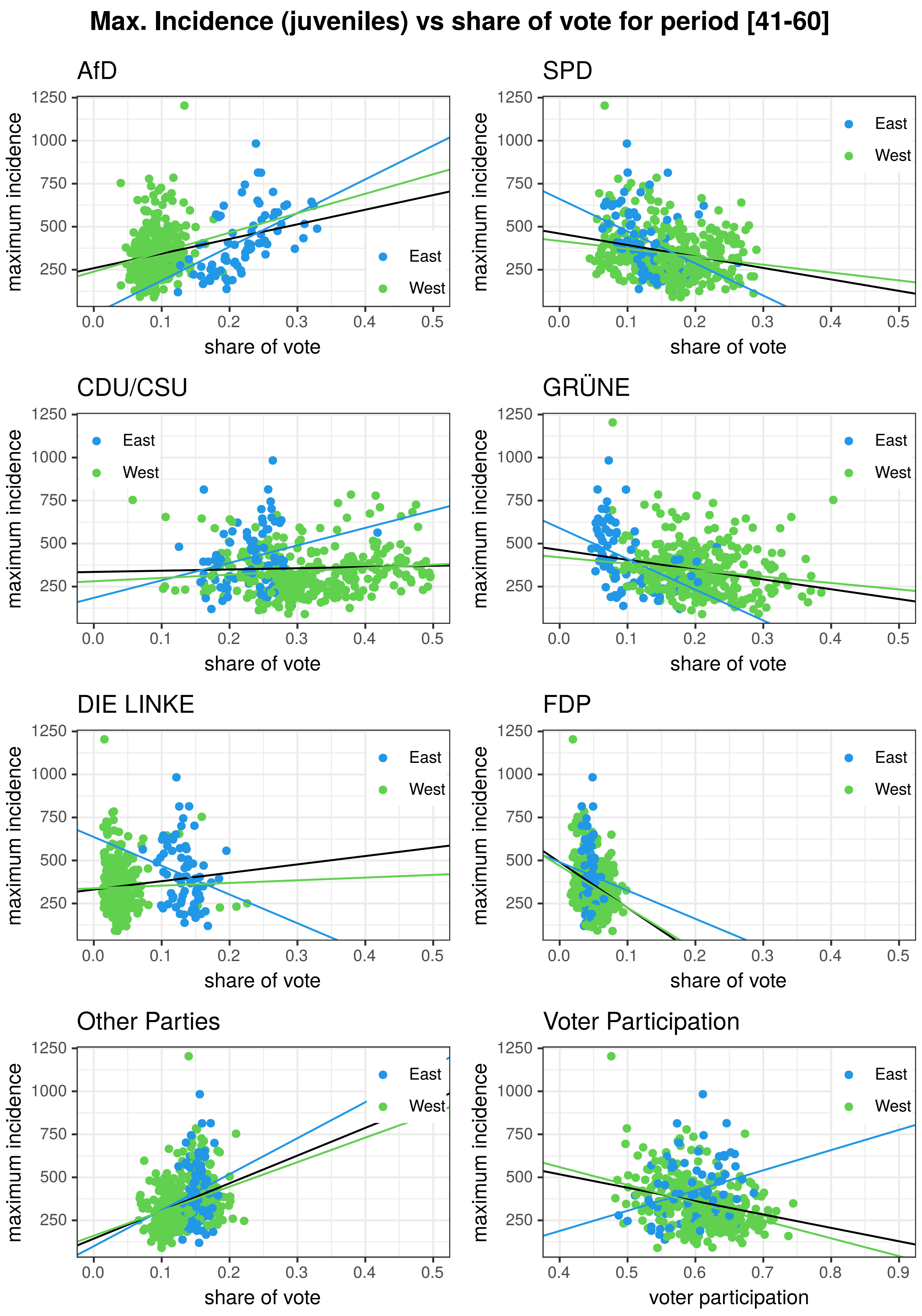
Max. Incidence (juveniles) vs share of vote for period [41-60]
AfD
SPD
CDU/CSU
GRÜNE
DIE LINKE
FDP
Other Parties
Voter Participation
East
West
maximum incidence
share of vote
voter participation
1250
1000
750
500
250
0.0
0.1
0.2
0.3
0.4
0.5
0.6
0.7
0.8
0.9

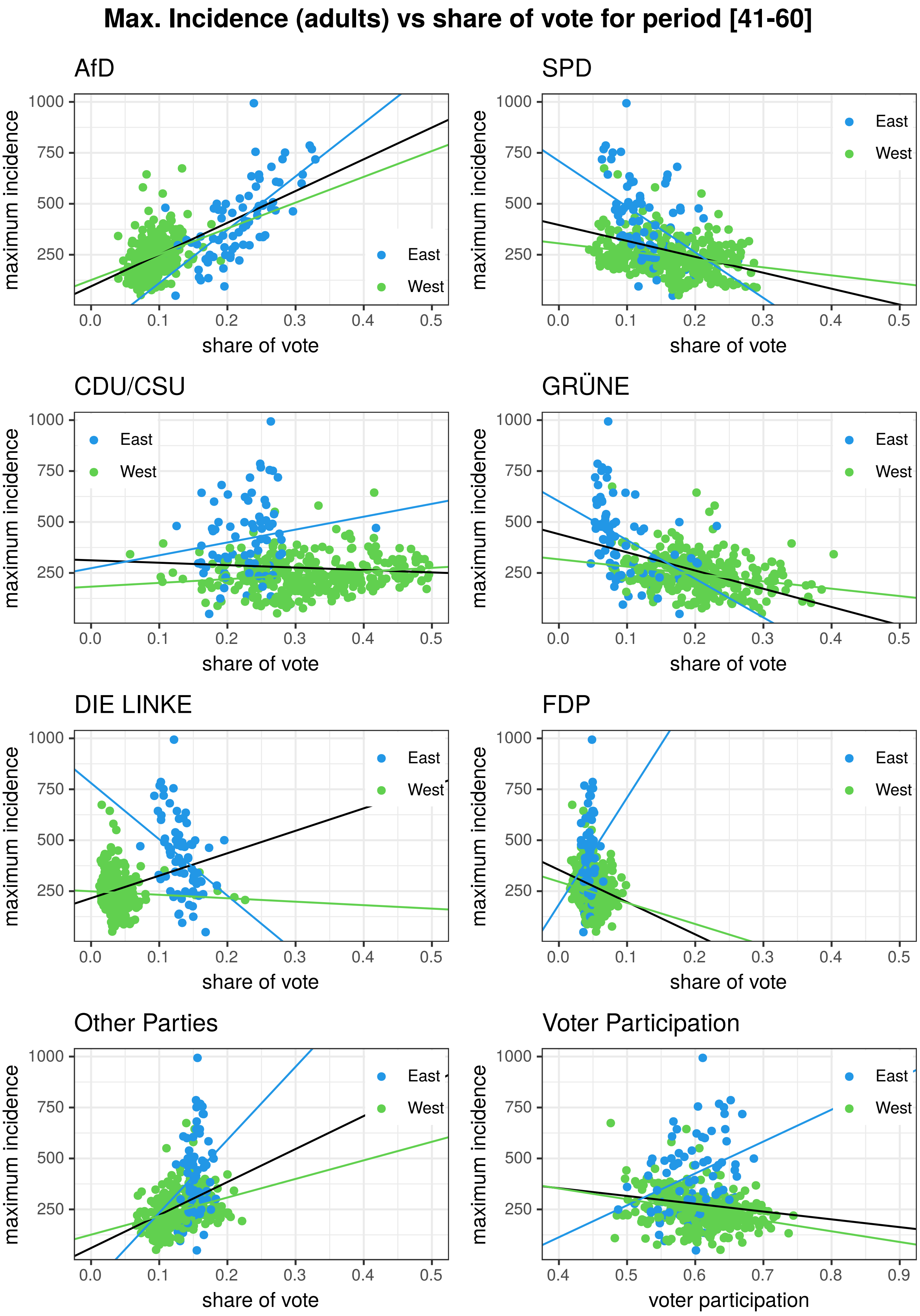

Max. Incidence (adults) vs share of vote for period [41-60]
AfD
SPD
CDU/CSU
GRÜNE
DIE LINKE
FDP
Other Parties
Voter Participation
maximum incidence
share of vote
voter participation
East
West

Max. Incidence (kids) vs share of vote for period [61-80]

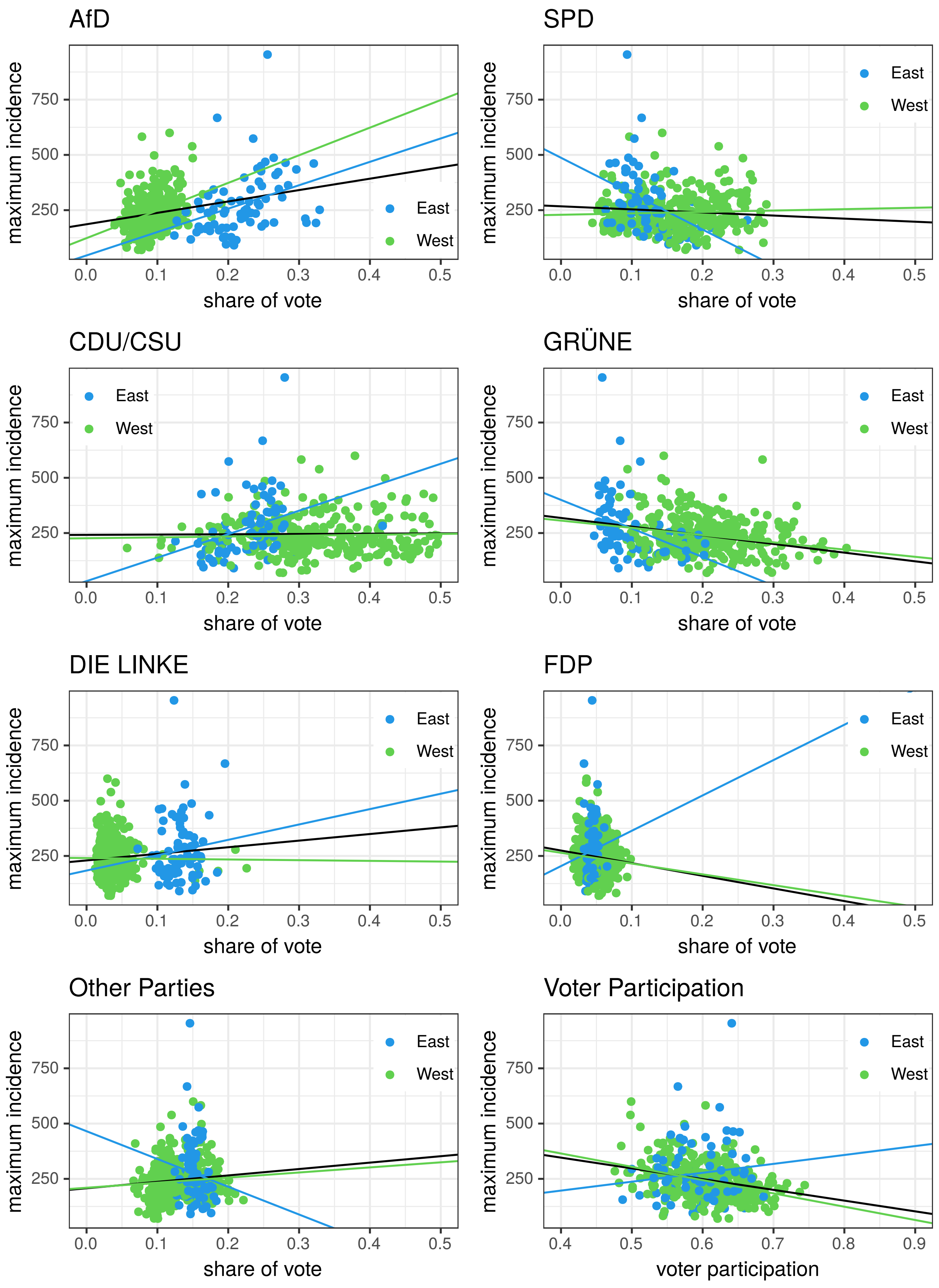

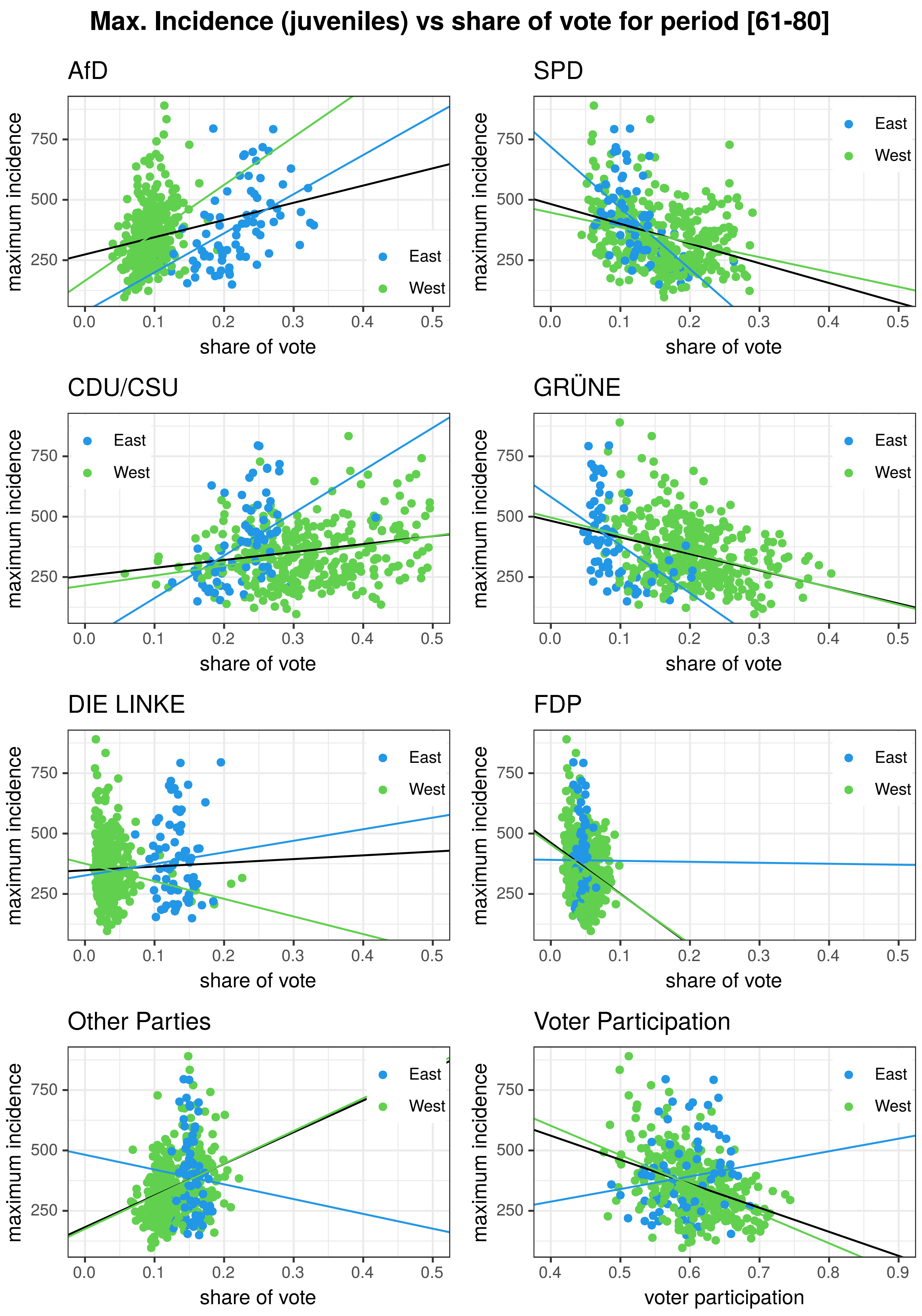
Max. Incidence (juveniles) vs share of vote for period [61-80]
AfD
SPD
CDU/CSU
GRÜNE
DIE LINKE
FDP
Other Parties
Voter Participation
maximum incidence
share of vote
voter participation
East
West
250
500
750
0.0
0.1
0.2
0.3
0.4
0.5
0.6
0.7
0.8
0.9

Max. Incidence (adults) vs share of vote for period [61-80]

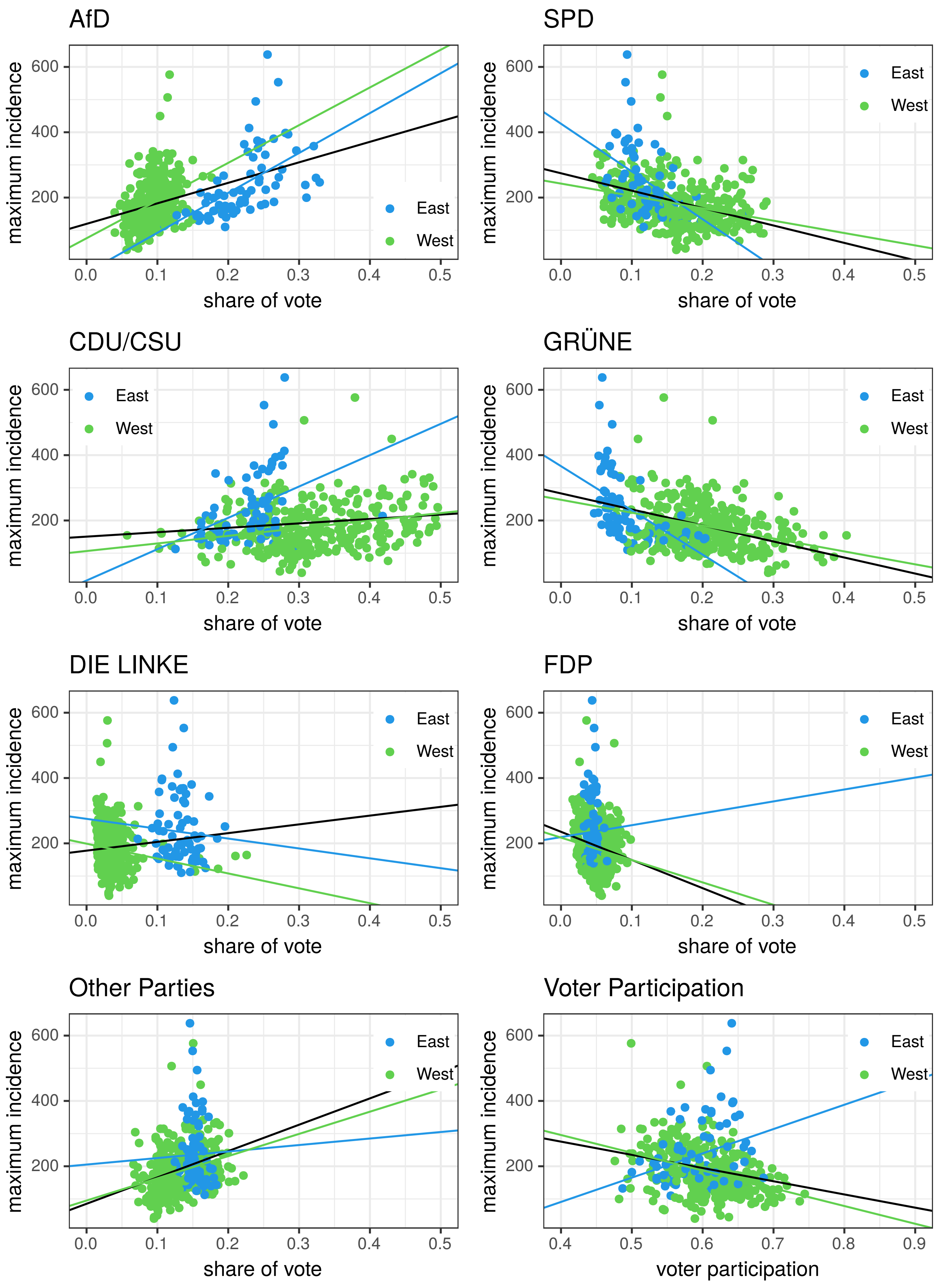

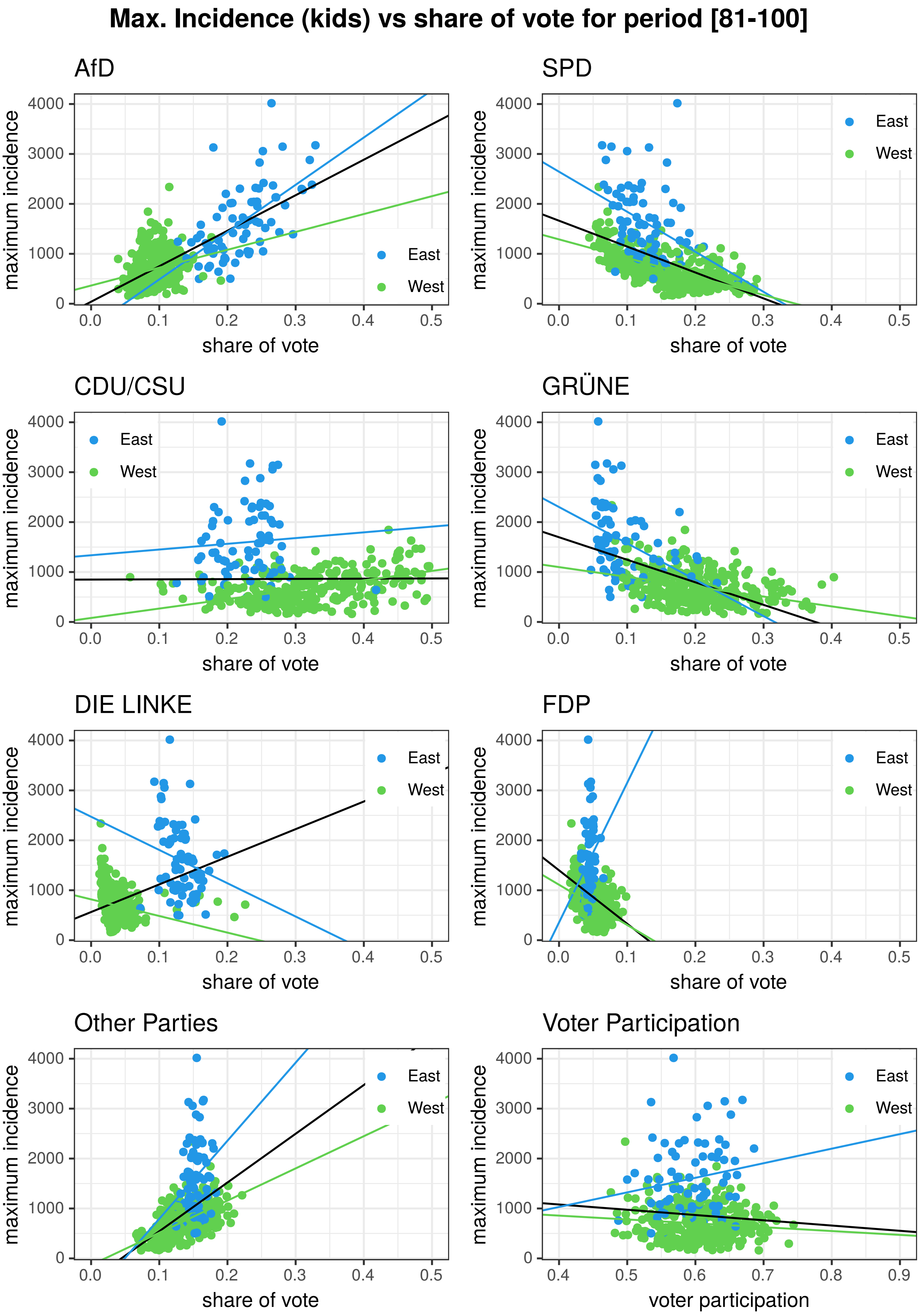
Max. Incidence (kids) vs share of vote for period [81-100]
AfD
SPD
CDU/CSU
GRÜNE
DIE LINKE
FDP
Other Parties
Voter Participation
maximum incidence
share of vote
voter participation
East
West
0
1000
2000
3000
4000
0.0
0.1
0.2
0.3
0.4
0.5
0.6
0.7
0.8
0.9

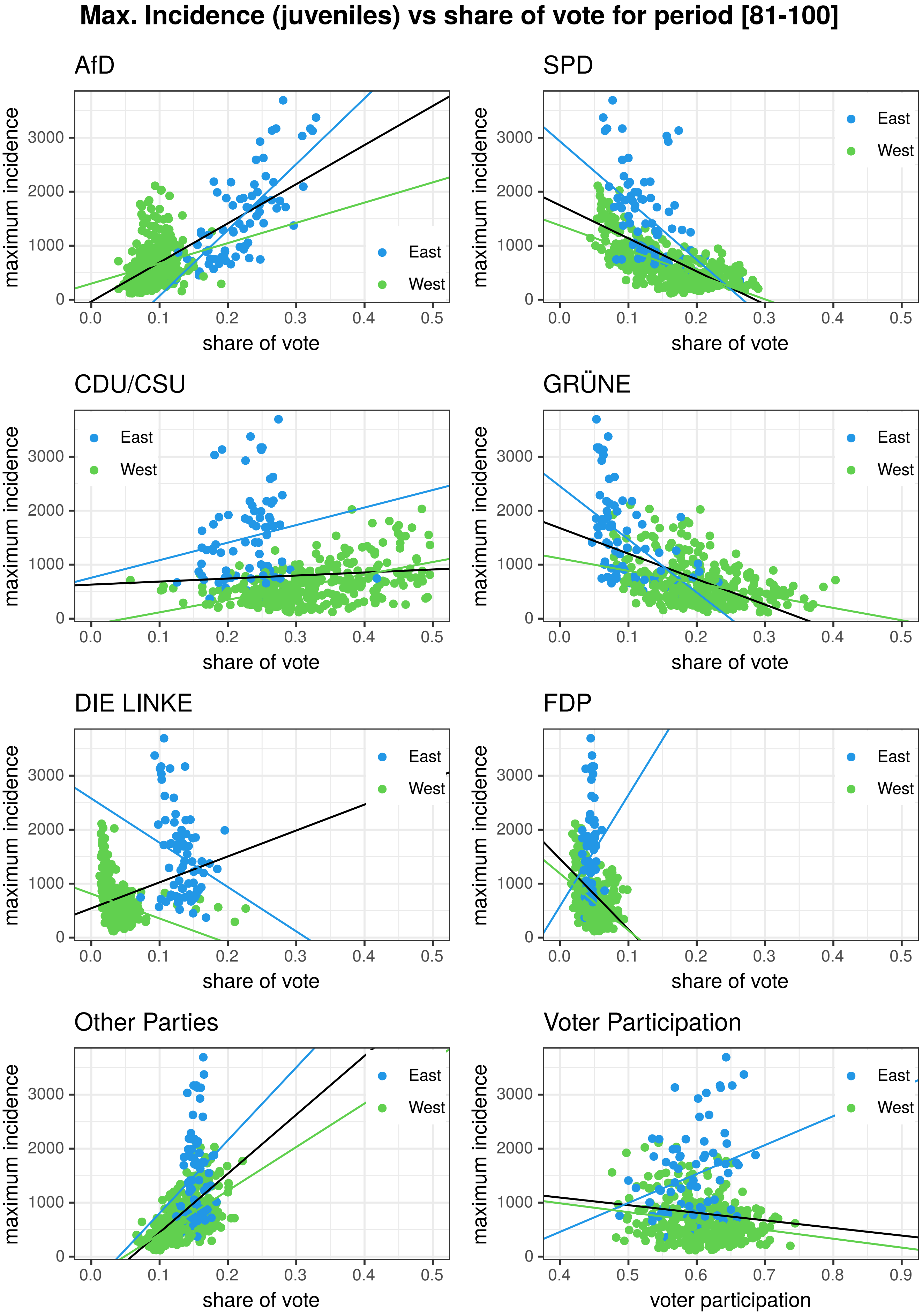

Max. Incidence (juveniles) vs share of vote for period [81-100]
AfD
SPD
CDU/CSU
GRÜNE
DIE LINKE
FDP
Other Parties
Voter Participation
maximum incidence
share of vote
voter participation
East
West

Max. Incidence (adults) vs share of vote for period [81-100]

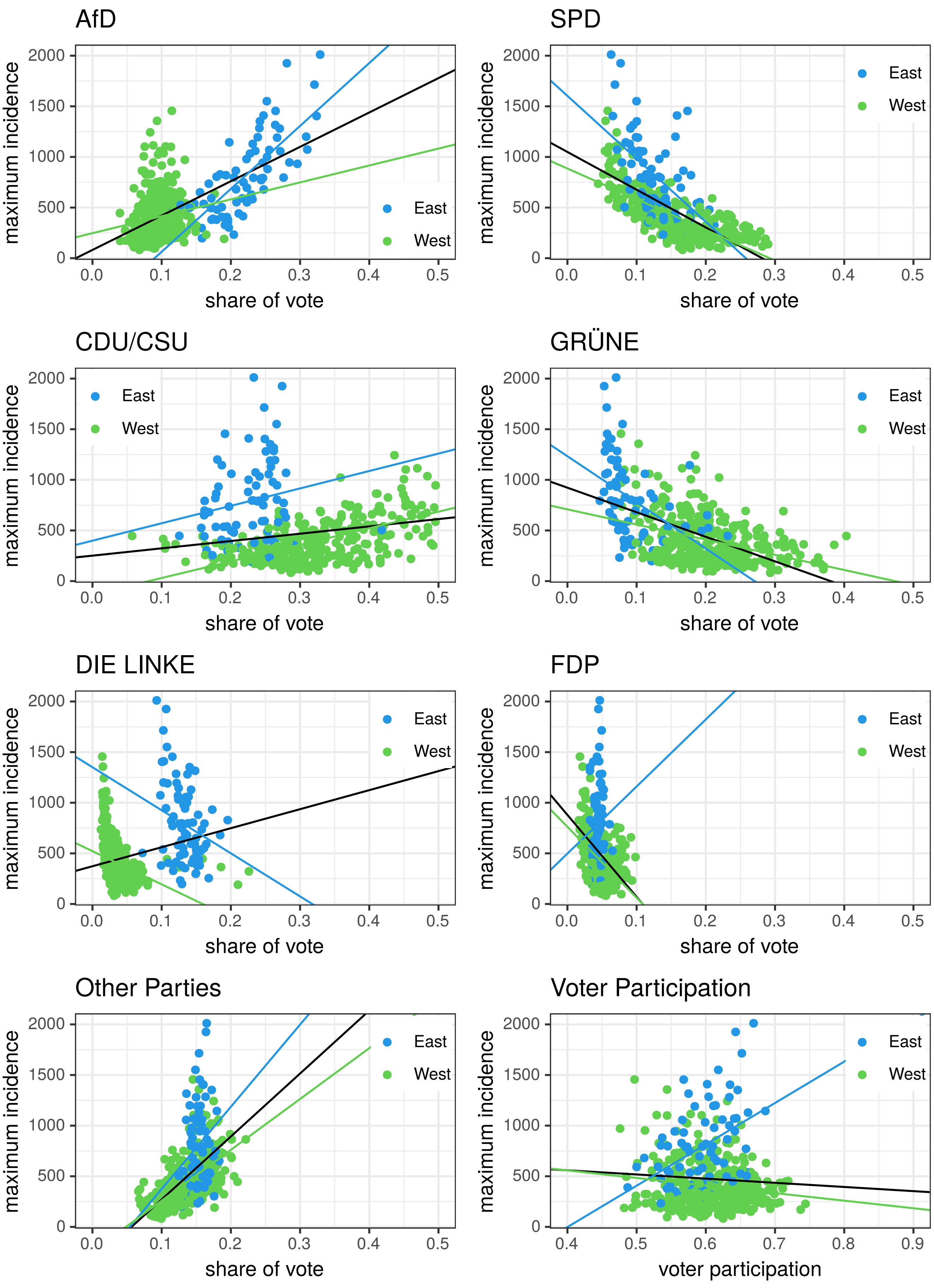

Supplement: Supplementary file 1 [file Data_Sheet_1.PDF]
