# Supplementary material for: Local socio-structural predictors of COVID-19 incidence in Germany
Source: Front Public Health. 2022 Sep 29;10:970092. doi: 10.3389/fpubh.2022.970092 (PMC9556738; doi:10.3389/fpubh.2022.970092)
Supplement: Supplementary file 2 [file Data_Sheet_2.PDF]

Supplement S2 to:

Alisha I. Qamar, Leonie Gronwald, Nina Timmesfeld, and Hans H. Diebner: Local Sociostructural Predictors of COVID-19 Incidence in Germany

The following series of figures shows age-stratified (3 age classes, *age*) locally observed **cumulative** incidences of 3 pre-defined epidemic periods (*p*) versus the share of vote (2019 European Parliament election) of 7 political parties and voter participation (*party*), respectively. Specifically, each panel depicts a scatterplot of cumulative incidence for a pair [*p*, *age*] by share of vote of a selected *party* for 411 German rural districts. Data points corresponding to East German districts are depicted in blue, West German districts in green. Three linear regression lines are shown for the full set of points (black), only the East German (blue), and only the West German (green) parts, respectively.
The header of each page contains the given pair [*p*, *age*], 9 in total, and each panel is labeled by the name of the selected *party*.

Cum. Incidence (kids) vs share of vote for period [41-60]

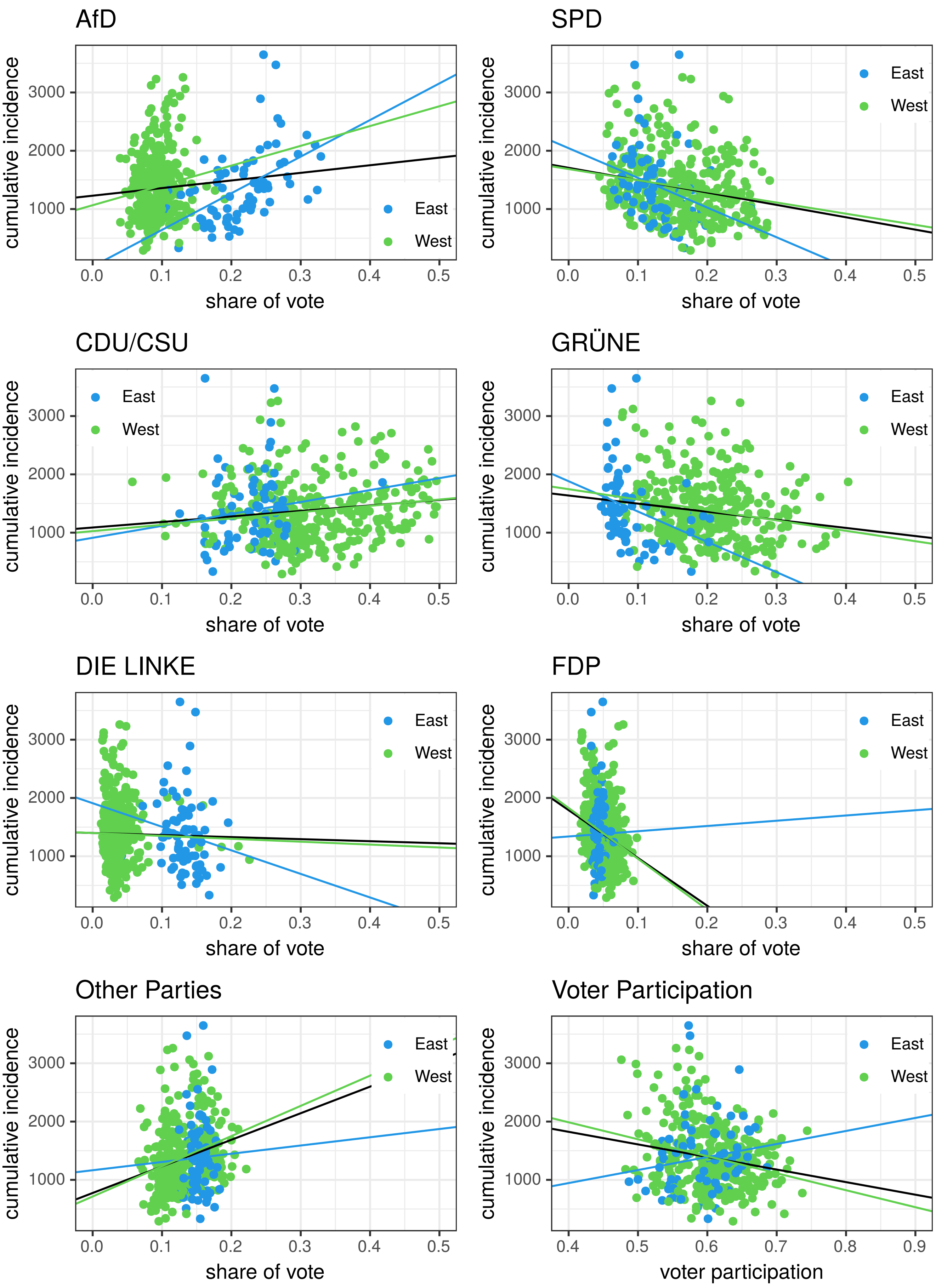

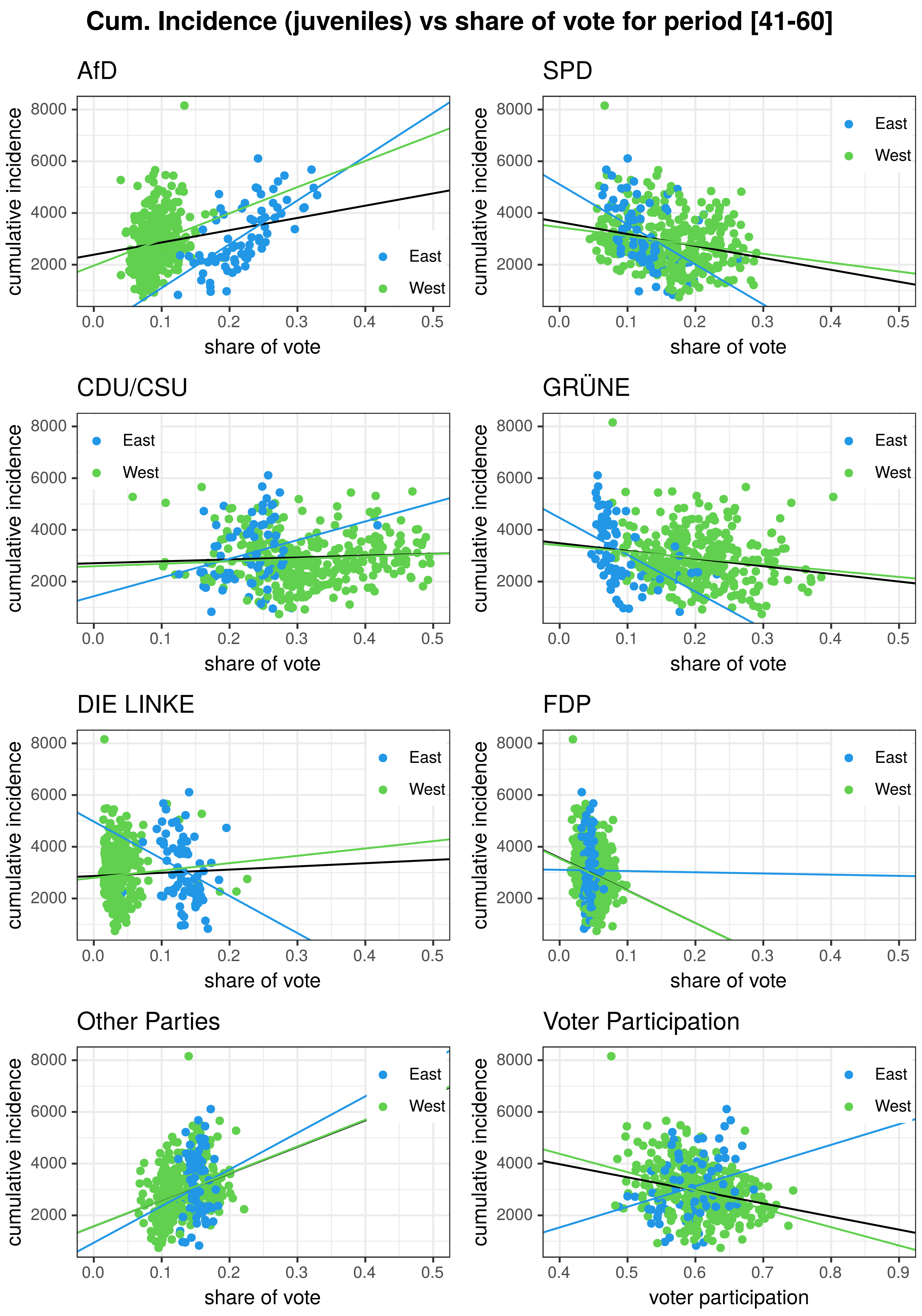
Cum. Incidence (juveniles) vs share of vote for period [41-60]
AfD
SPD
CDU/CSU
GRÜNE
DIE LINKE
FDP
Other Parties
Voter Participation
cumulative incidence
share of vote
voter participation
East
West
0.0
0.1
0.2
0.3
0.4
0.5
0.6
0.7
0.8
0.9
2000
4000
6000
8000

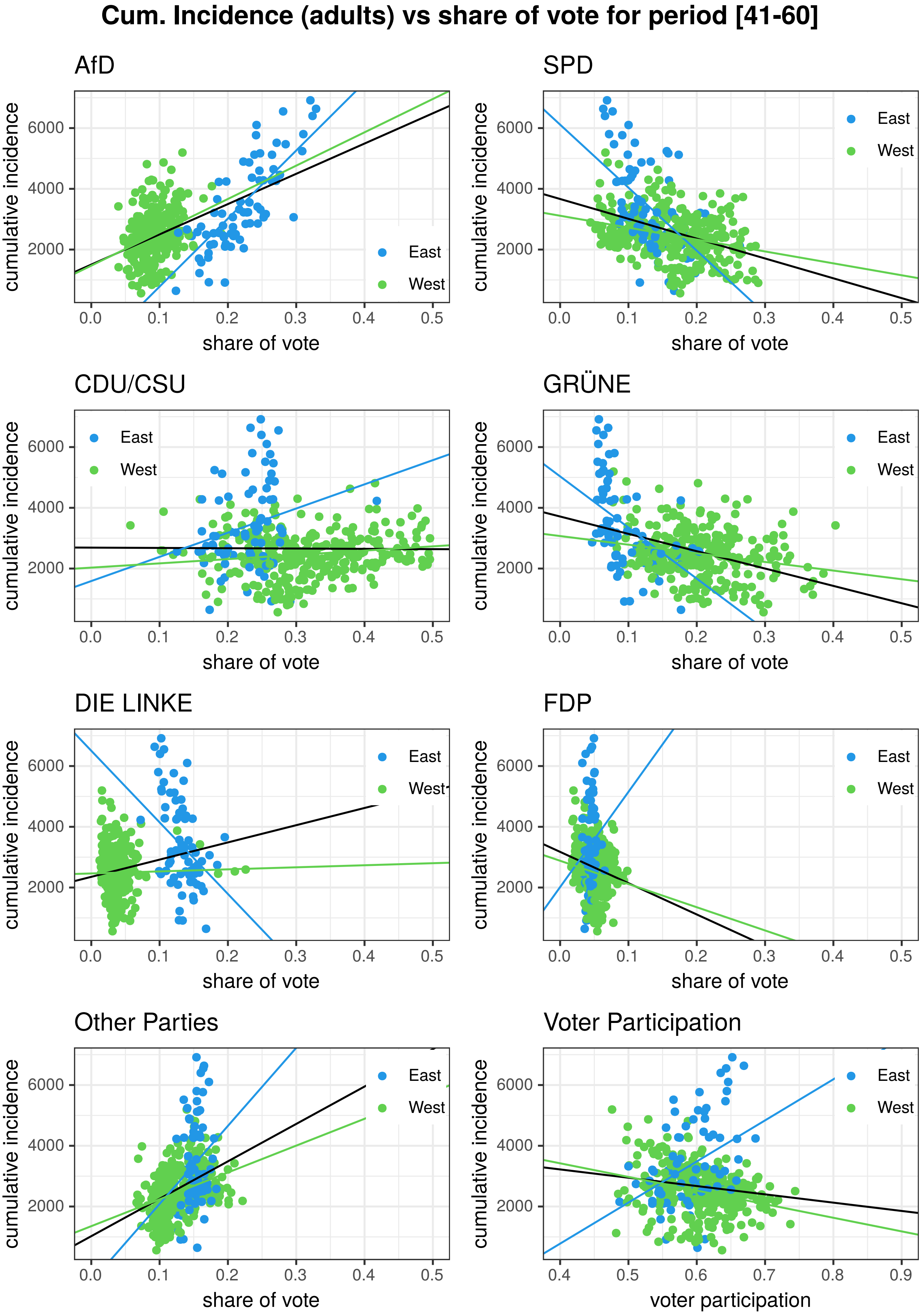
Cum. Incidence (adults) vs share of vote for period [41-60]
AfD
SPD
CDU/CSU
GRÜNE
DIE LINKE
FDP
Other Parties
Voter Participation
cumulative incidence
share of vote
voter participation
East
West
0.0
0.1
0.2
0.3
0.4
0.5
0.6
0.7
0.8
0.9
2000
4000
6000

Cum. Incidence (kids) vs share of vote for period [61-80]

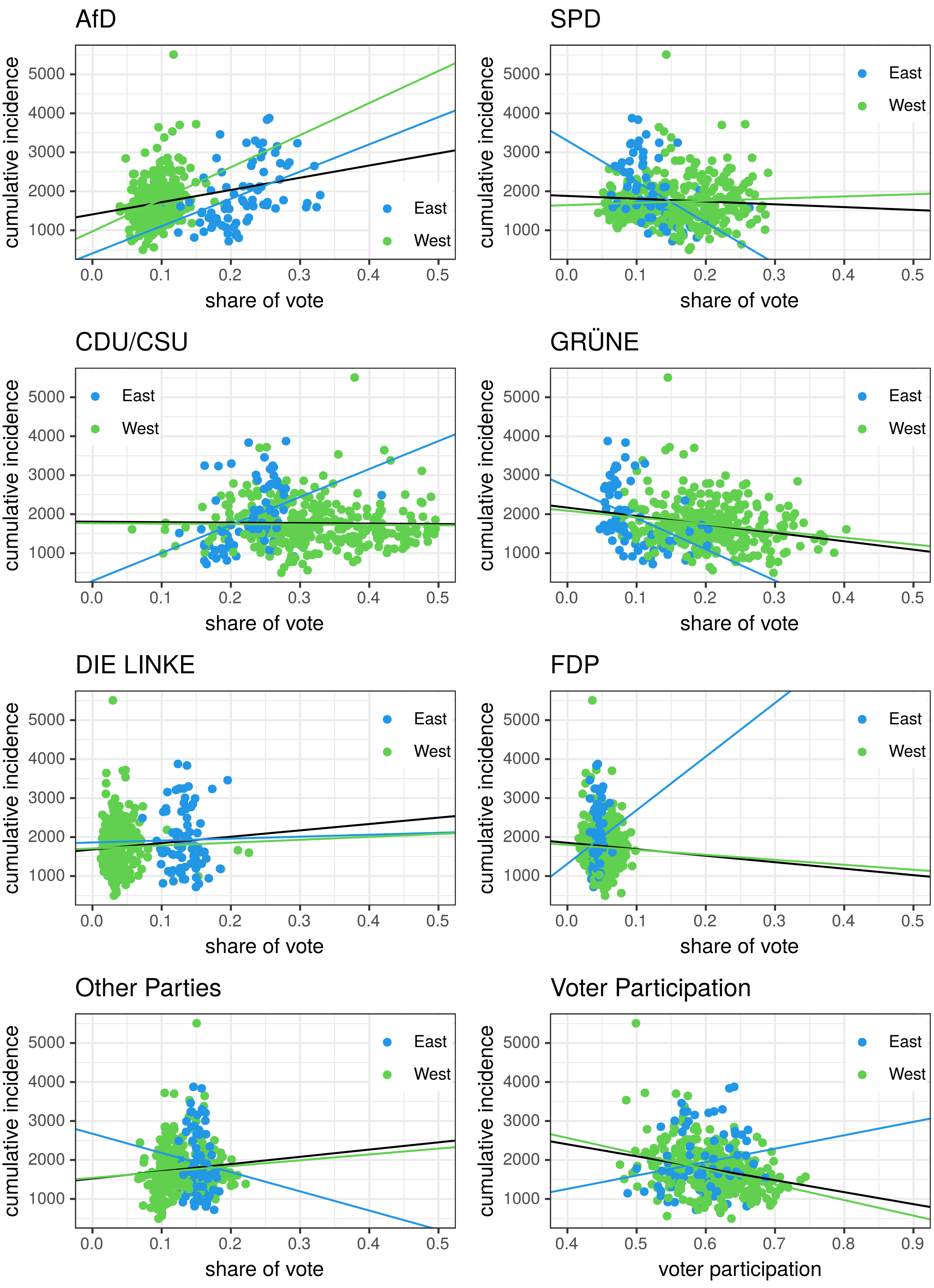

Cum. Incidence (juveniles) vs share of vote for period [61-80]

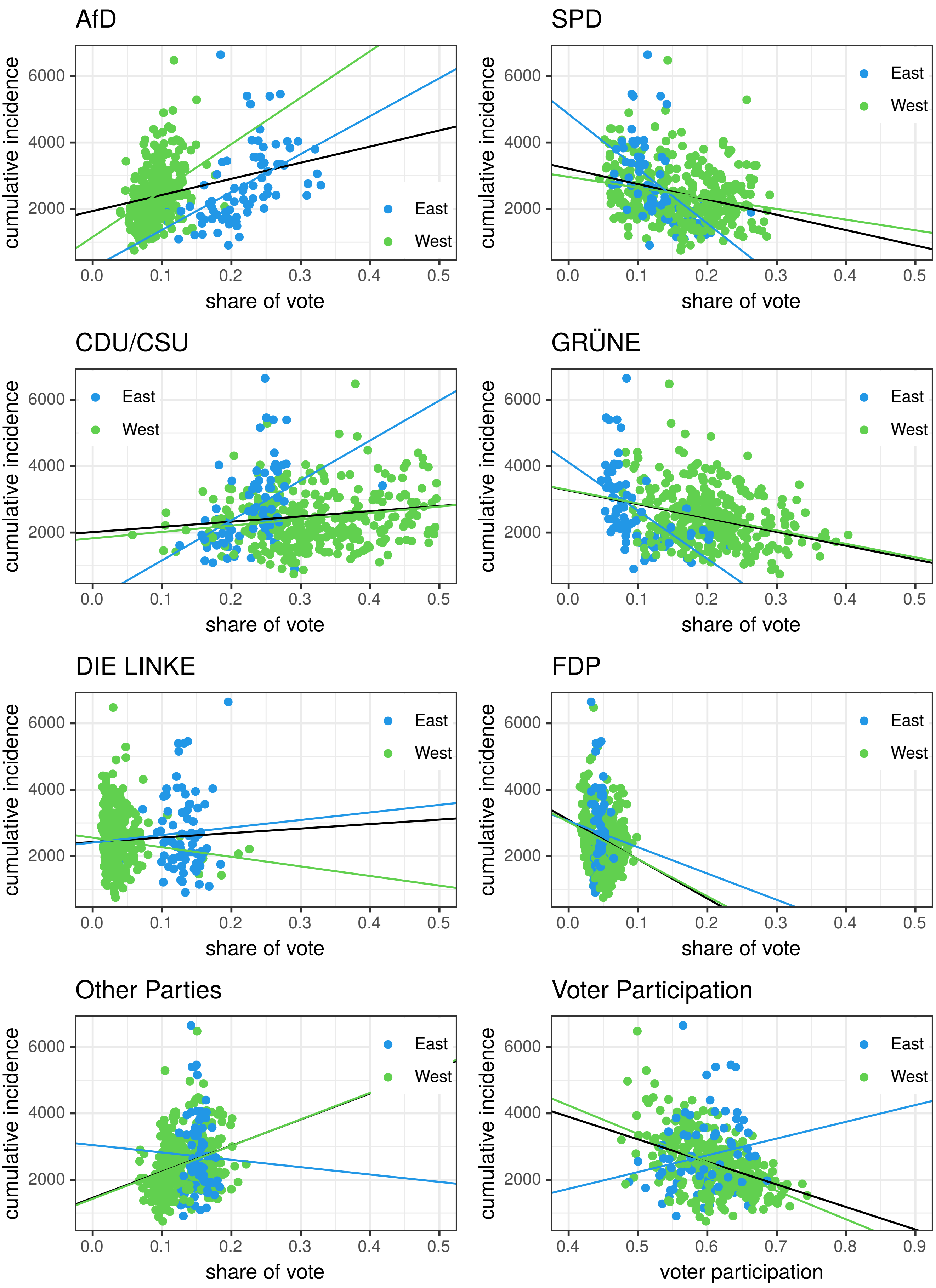

Cum. Incidence (adults) vs share of vote for period [61-80]

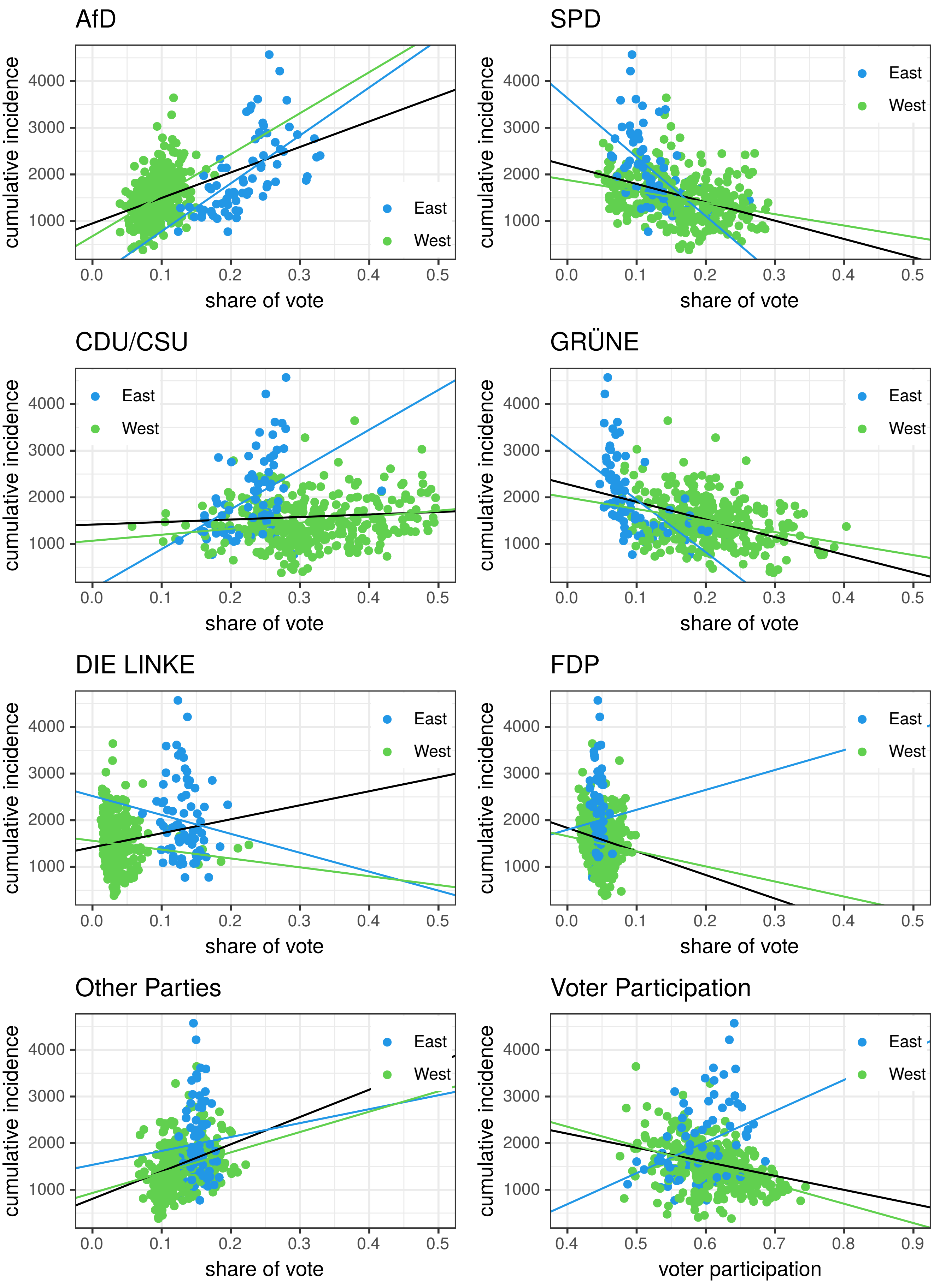

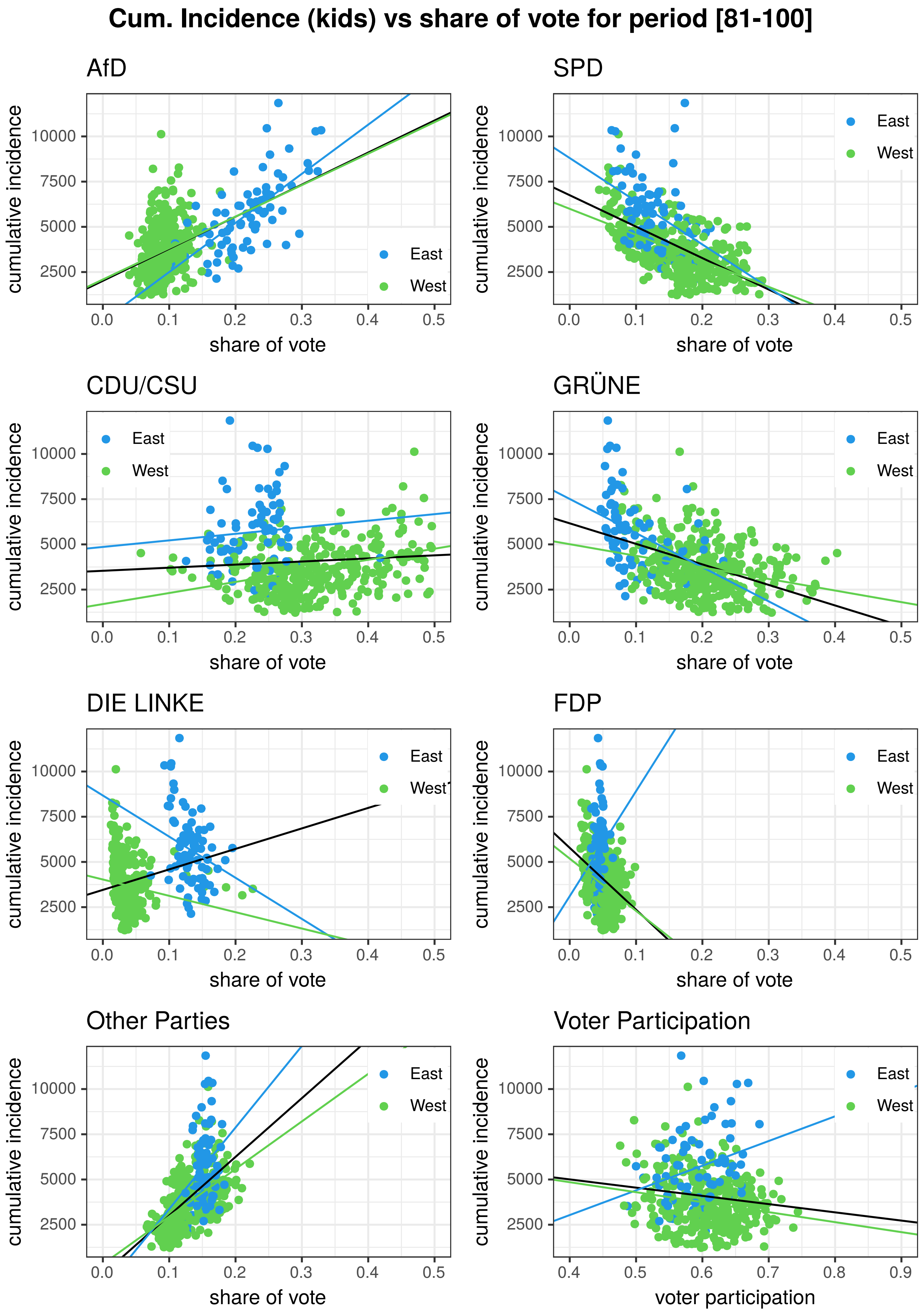
Cum. Incidence (kids) vs share of vote for period [81-100]
AfD
SPD
CDU/CSU
GRÜNE
DIE LINKE
FDP
Other Parties
Voter Participation
cumulative incidence
2500
5000
7500
10000
share of vote
0.0
0.1
0.2
0.3
0.4
0.5
voter participation
0.4
0.5
0.6
0.7
0.8
0.9
East
West

Cum. Incidence (juveniles) vs share of vote for period [81-100]

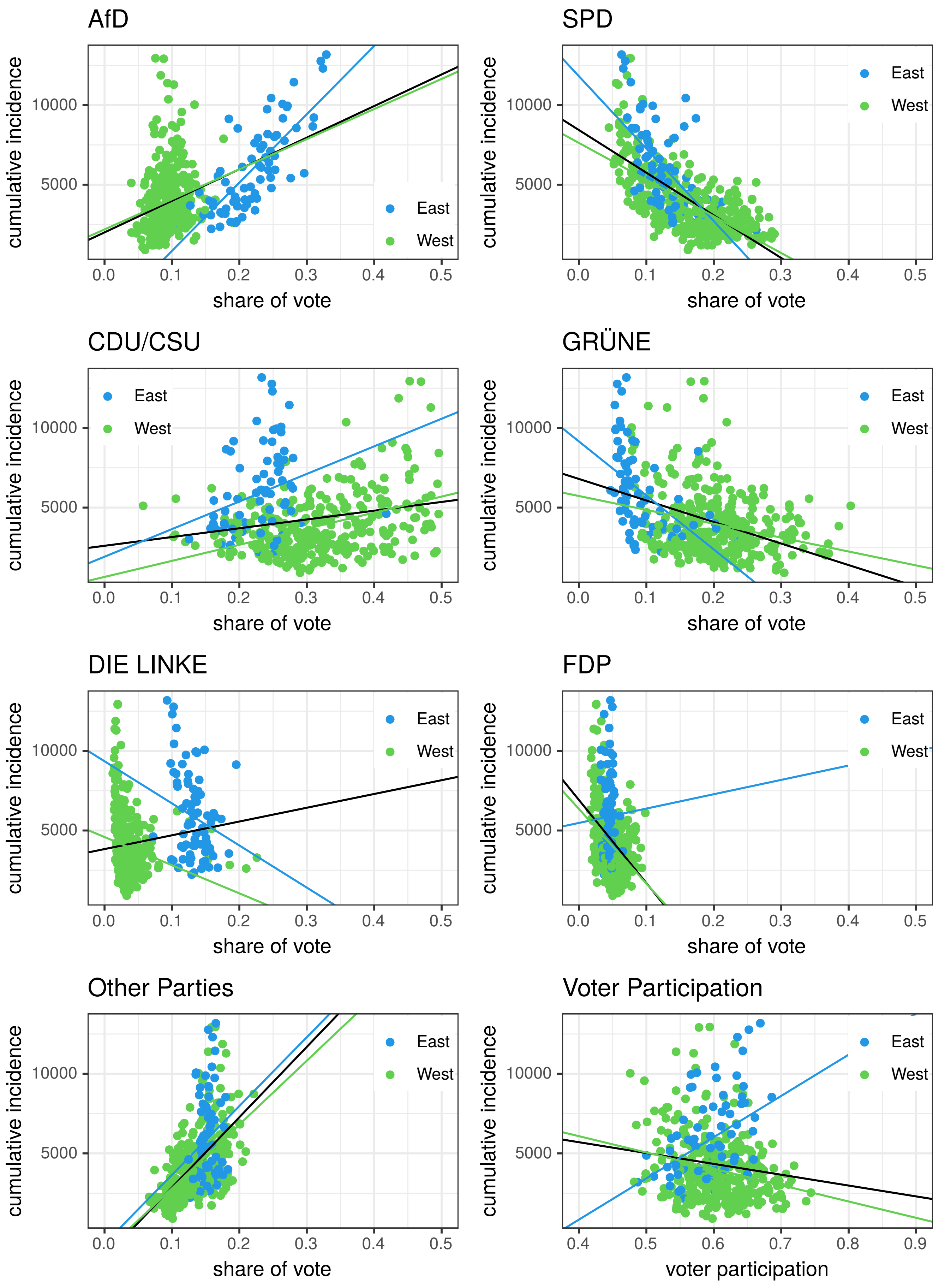

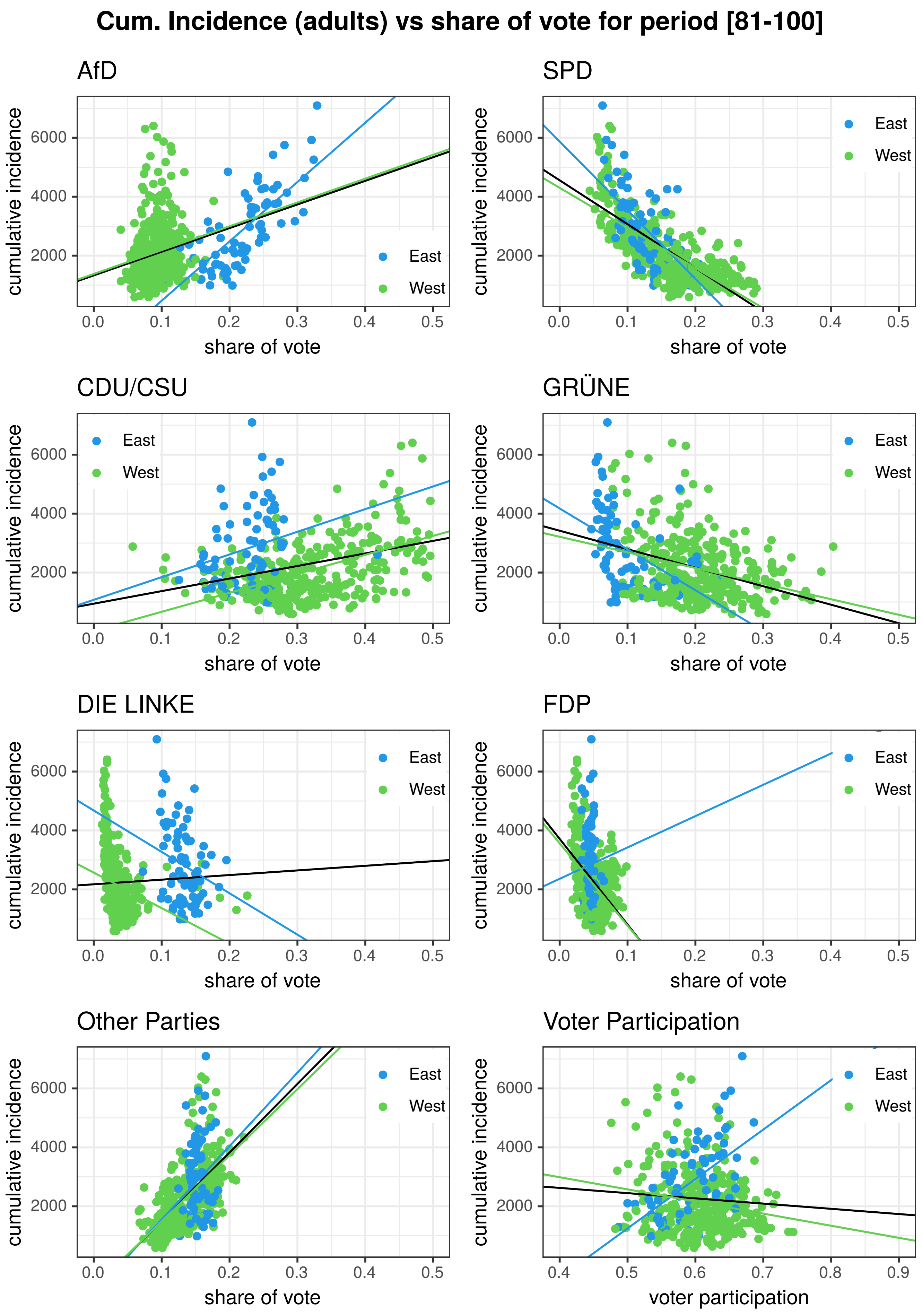
Cum. Incidence (adults) vs share of vote for period [81-100]
AfD
SPD
CDU/CSU
GRÜNE
DIE LINKE
FDP
Other Parties
Voter Participation
cumulative incidence
6000
4000
2000
0.0
0.1
0.2
0.3
0.4
0.5
share of vote
0.4
0.5
0.6
0.7
0.8
0.9
voter participation
East
West
