# Supplementary material for: Local socio-structural predictors of COVID-19 incidence in Germany
Source: Front Public Health. 2022 Sep 29;10:970092. doi: 10.3389/fpubh.2022.970092 (PMC9556738; doi:10.3389/fpubh.2022.970092)

Supplement S3 to:

Alisha I. Qamar, Leonie Gronwald, Nina Timmesfeld, and Hans H. Diebner: Local Sociostructural Predictors of COVID-19 Incidence in Germany

The following series of figures shows age-stratified (3 age classes, *age*) locally observed **cumulative** incidences of 3 pre-defined epidemic periods (*p*) versus eight socioeconomic/geopolitical parameters (*socioeco*), respectively. The eight parameters are:

- unemployment rate
- proportion of protection seekers
- proportion social benefit claimants
- per capita income
- proportion with higher education level
- proportion with middle education level
- proportion with low education level
- population density (inhabitants per square kilometre)

Specifically, each panel depicts a scatterplot of cumulative incidence for a pair [*p*, *age*] by one of the socioeconomic parameters (*socioeco*) for 411 German rural districts. Data points corresponding to East German districts are depicted in blue, West German districts in green. Three linear regression lines are shown for the full set of points (black), only the East German (blue), and only the West German (green) parts, respectively.

The header of each page contains the given pair [*p*, *age*], 9 in total, and each panel is labeled by the name of the selected *socioeco*.

Cum. Incidence (kids) vs socieconomic parameter [41-60]

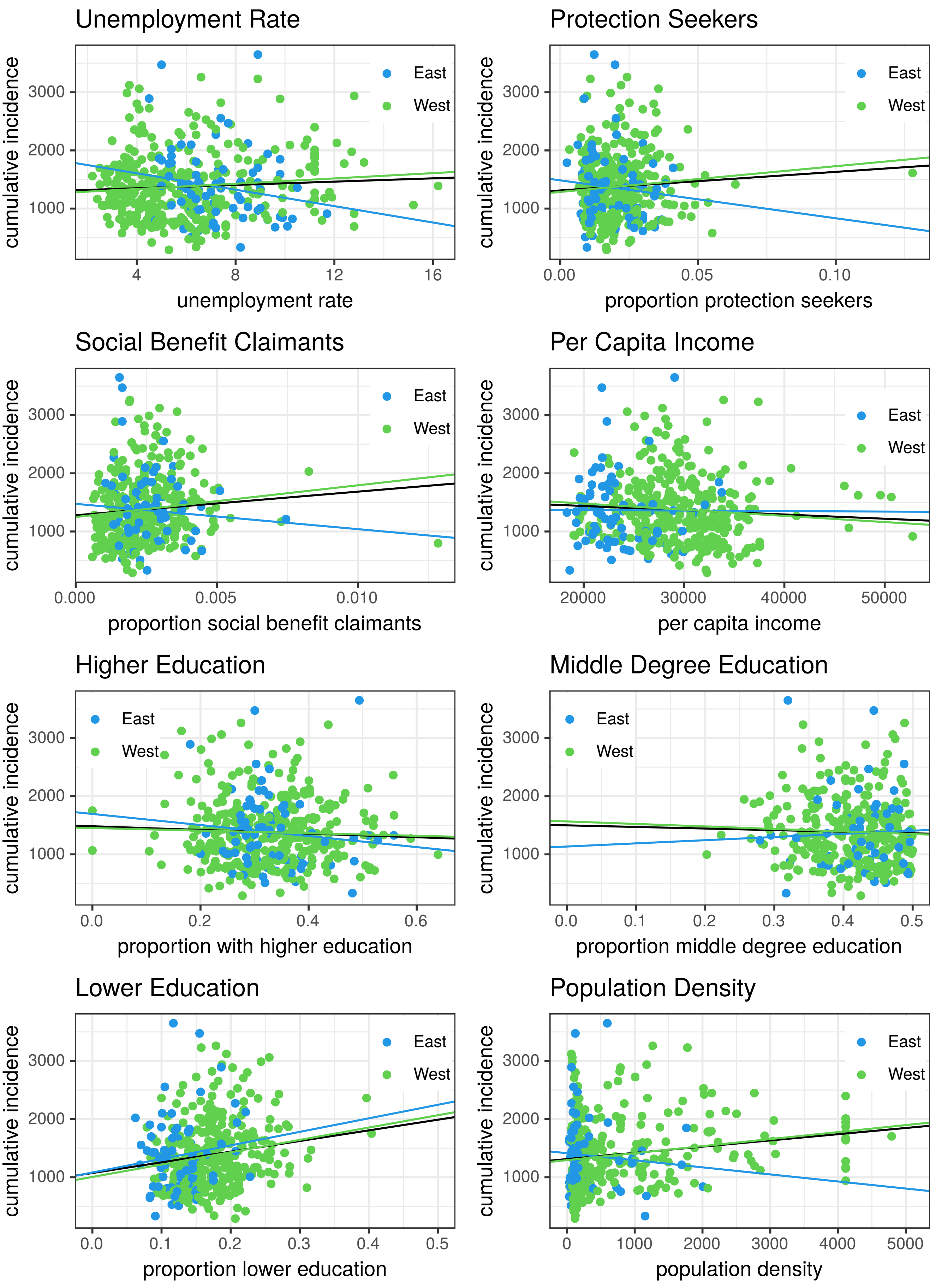

Cum. Incidence (juveniles) vs socieconomic parameter [41-60]

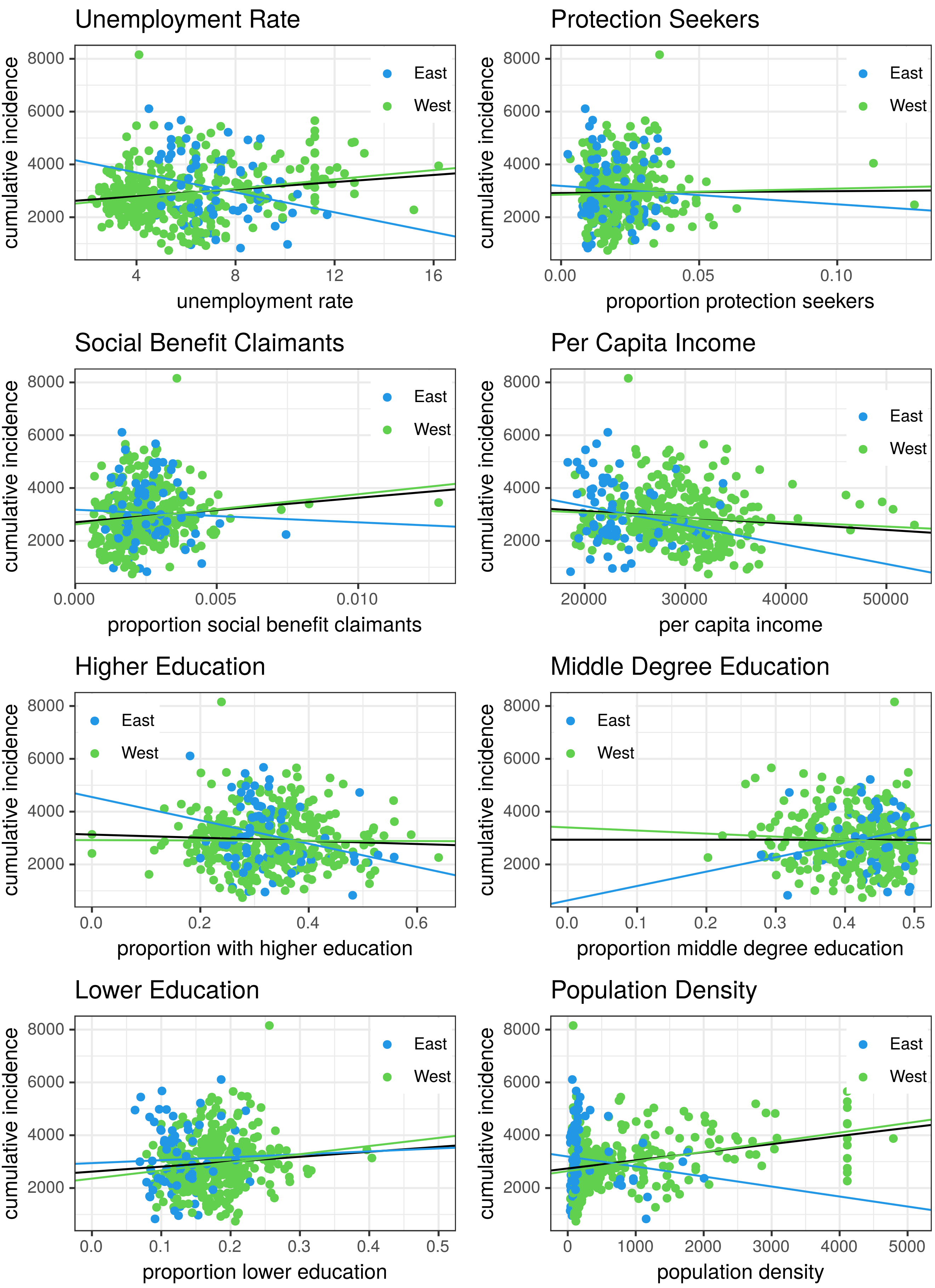

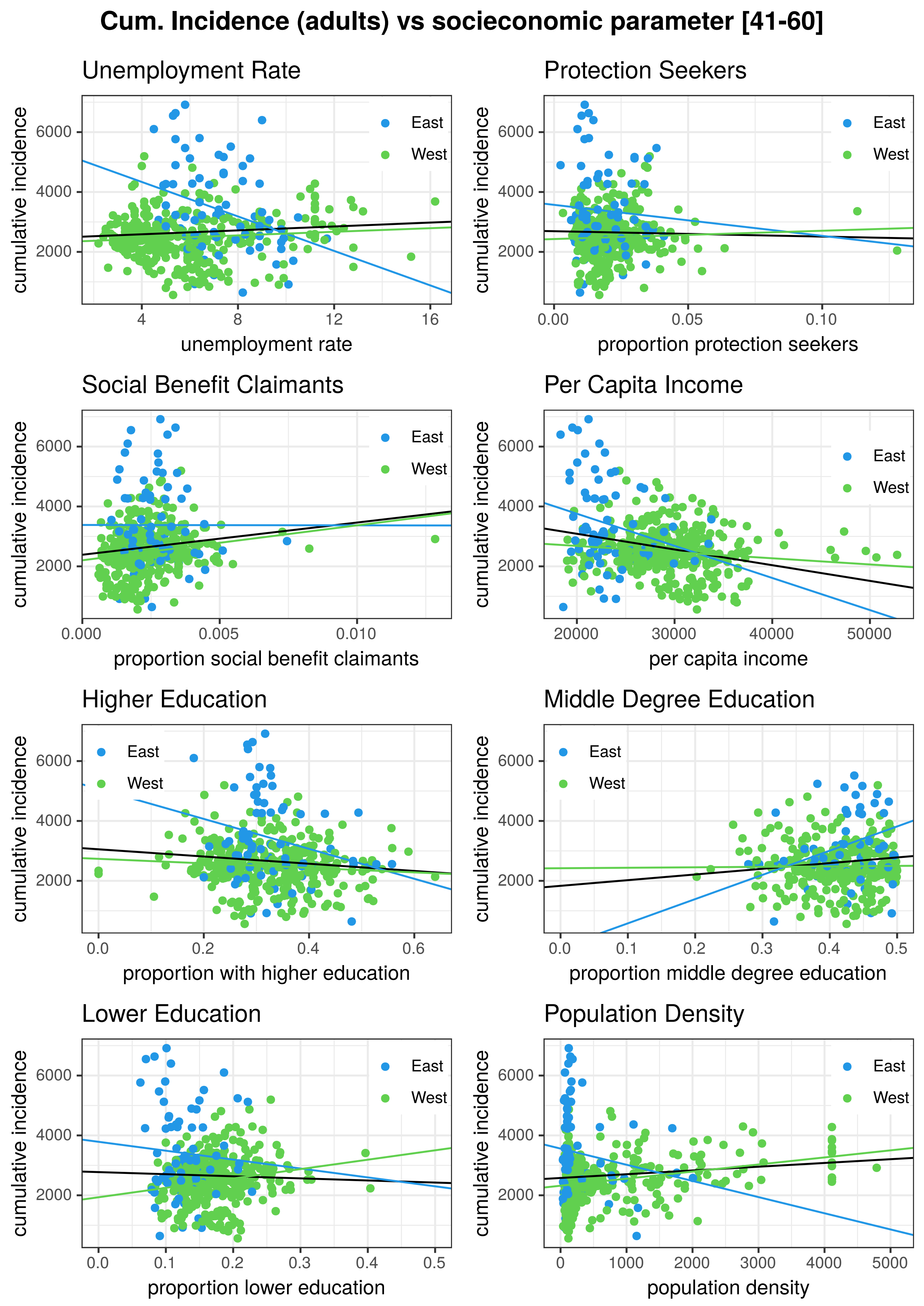

Cum. Incidence (adults) vs socieconomic parameter [41-60]
Unemployment Rate
cumulative incidence
unemployment rate
Protection Seekers
proportion protection seekers
Social Benefit Claimants
proportion social benefit claimants
Per Capita Income
per capita income
Higher Education
proportion with higher education
Middle Degree Education
proportion middle degree education
Lower Education
proportion lower education
Population Density
population density
East
West

Cum. Incidence (kids) vs socieconomic parameter [61-80]

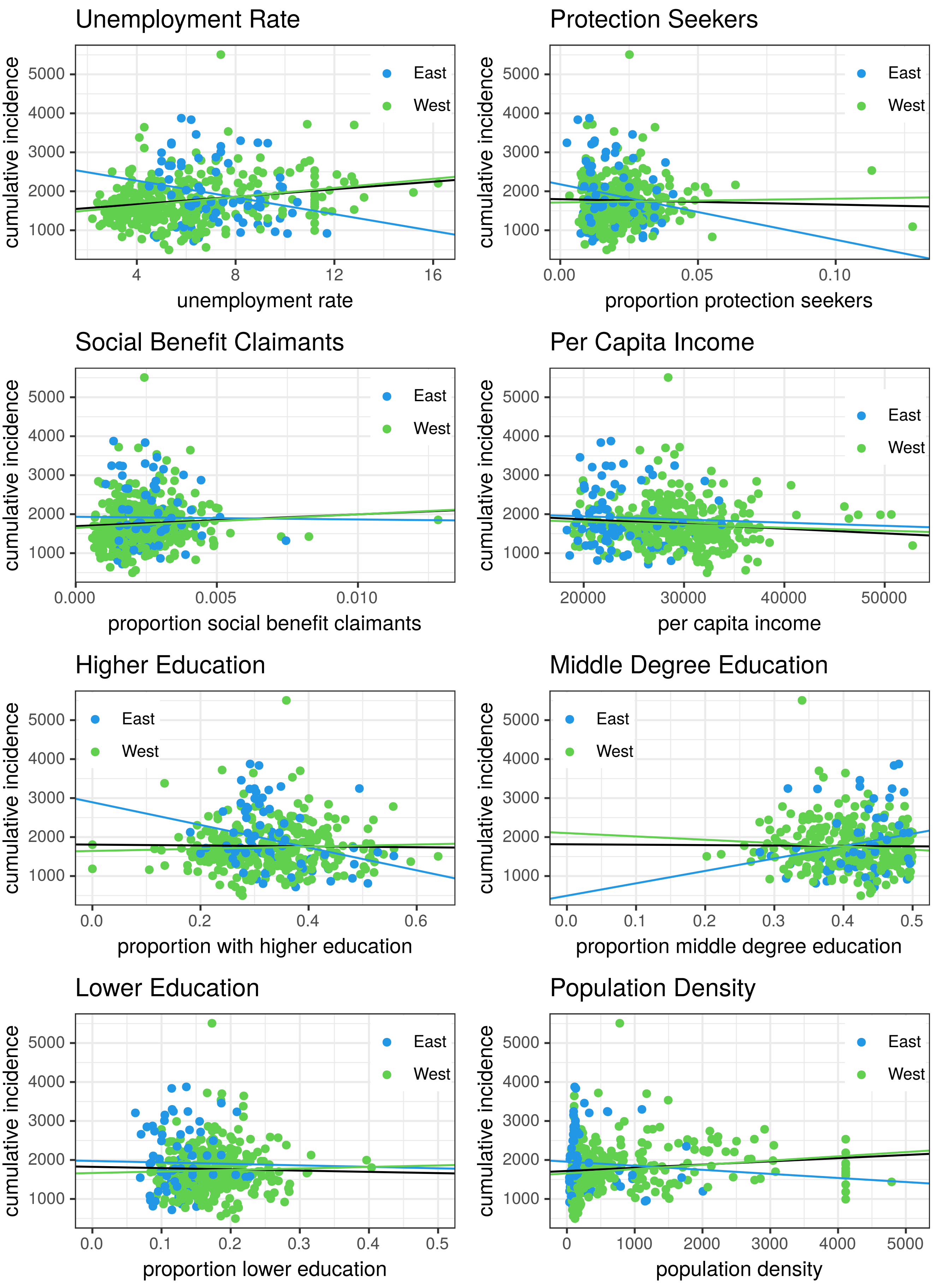

Cum. Incidence (juveniles) vs socieconomic parameter [61-80]

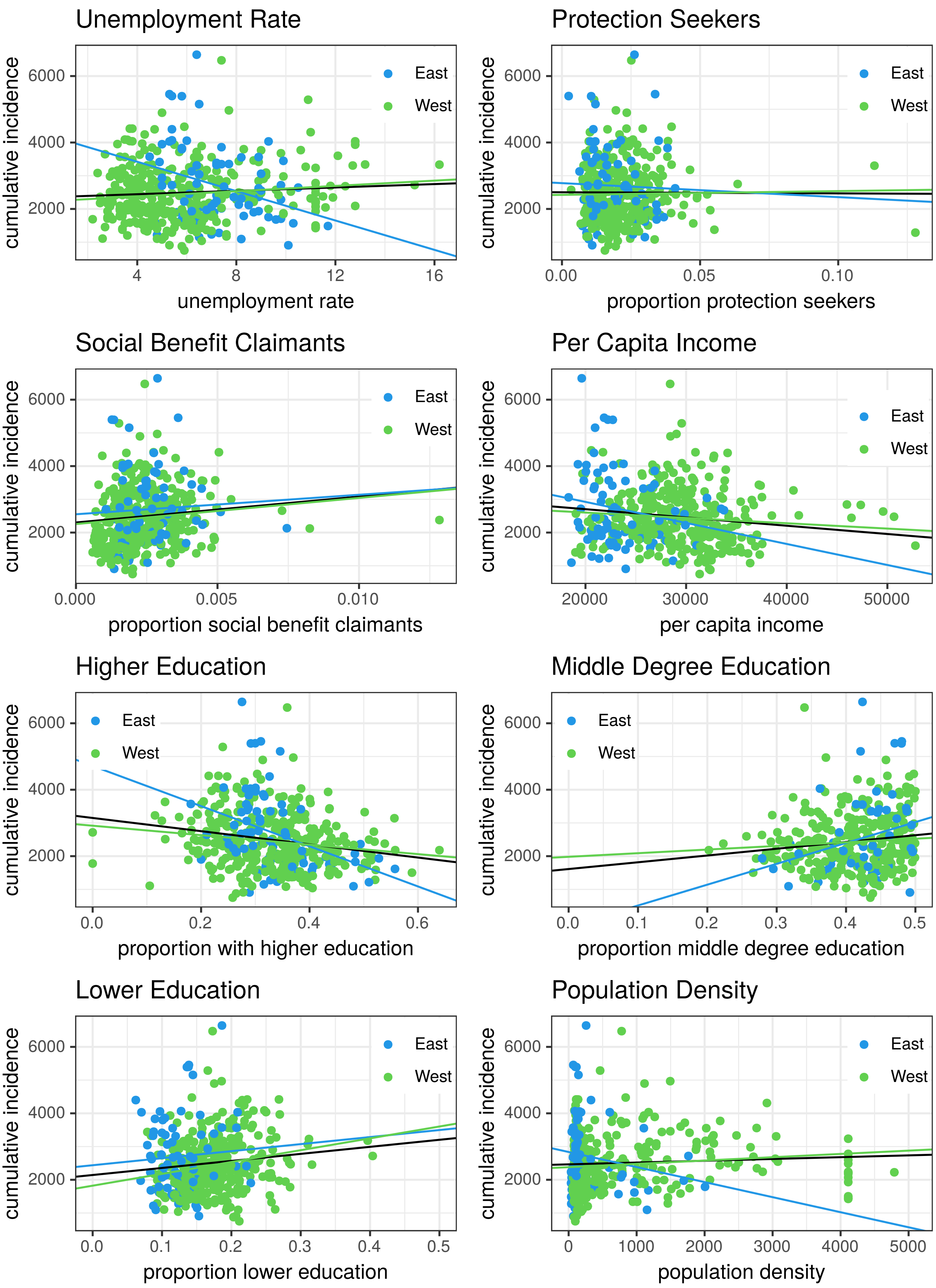

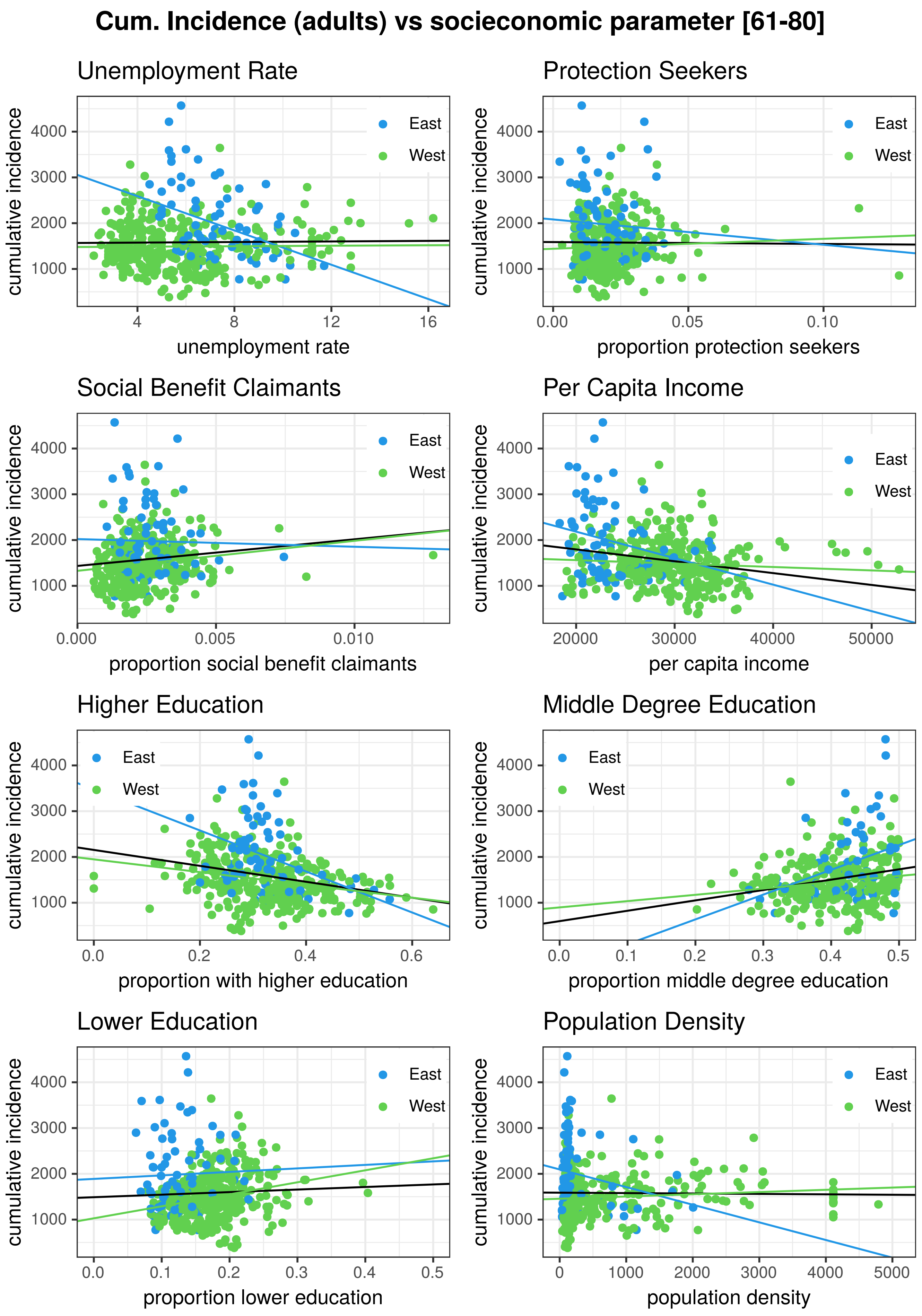

Cum. Incidence (adults) vs socieconomic parameter [61-80]
Unemployment Rate
cumulative incidence
unemployment rate
Protection Seekers
proportion protection seekers
Social Benefit Claimants
proportion social benefit claimants
Per Capita Income
per capita income
Higher Education
proportion with higher education
Middle Degree Education
proportion middle degree education
Lower Education
proportion lower education
Population Density
population density
East
West

Cum. Incidence (kids) vs socieconomic parameter [81-100]

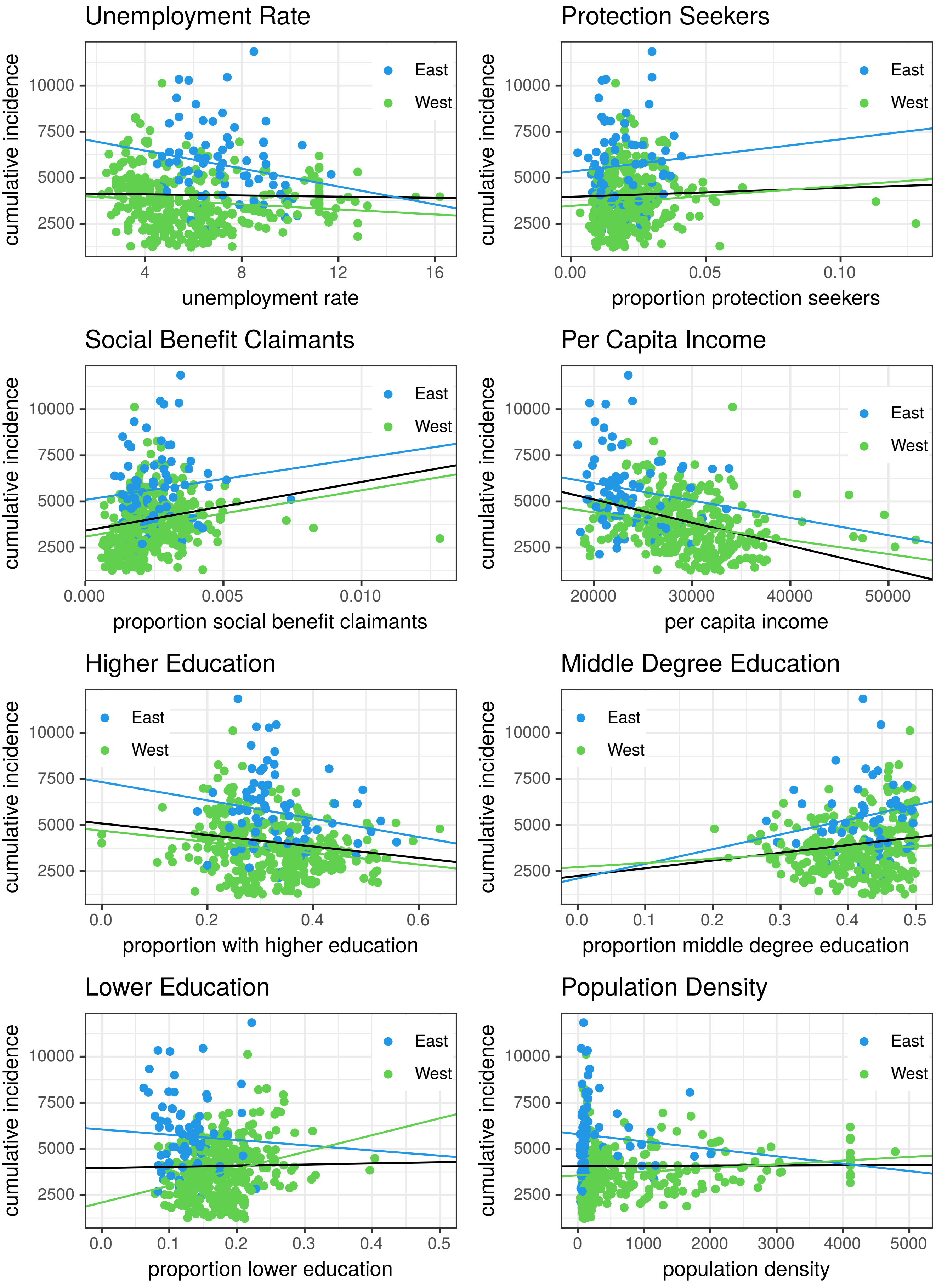

Cum. Incidence (juveniles) vs socieconomic parameter [81-100]

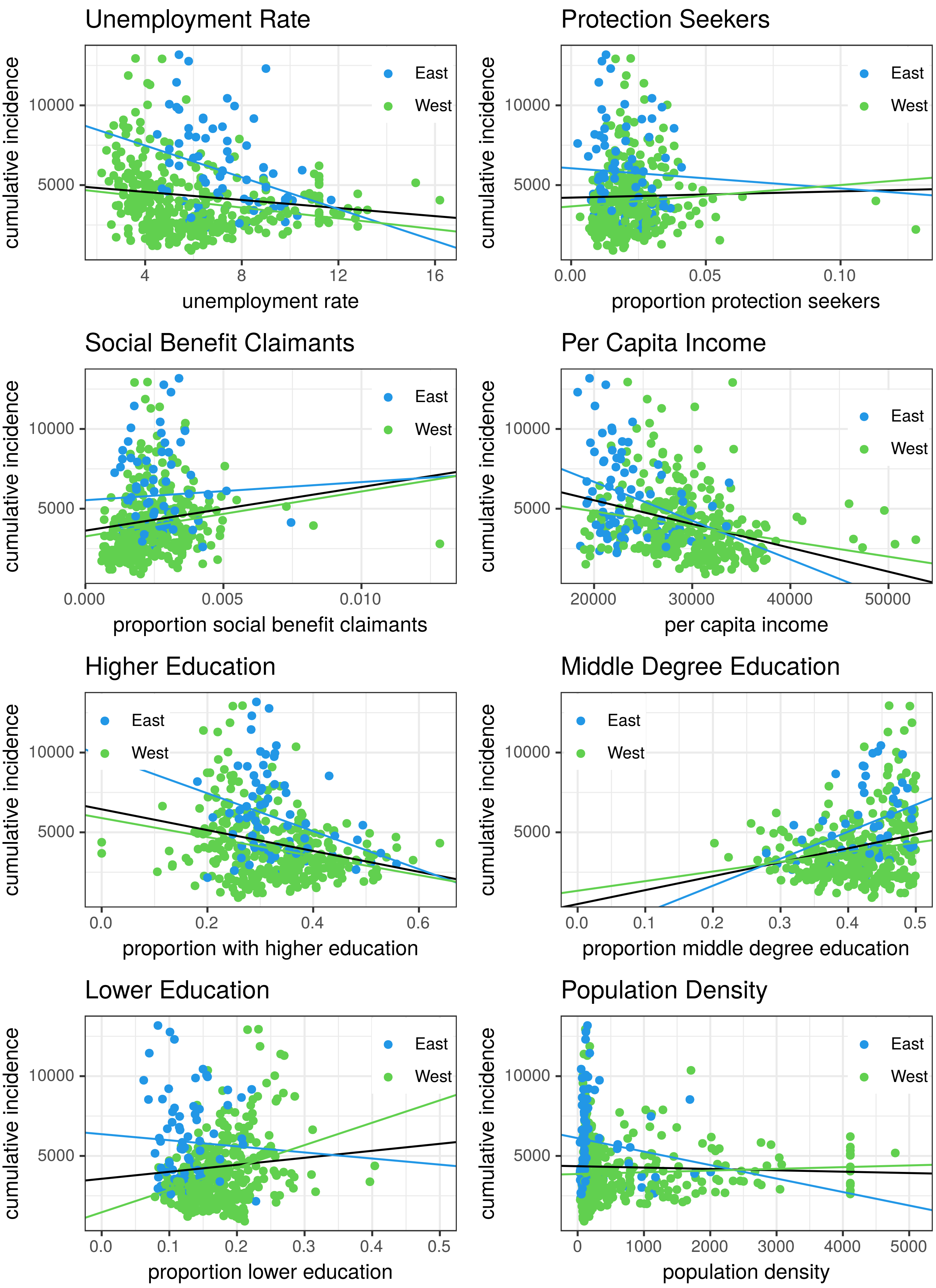

Cum. Incidence (adults) vs socieconomic parameter [81-100]

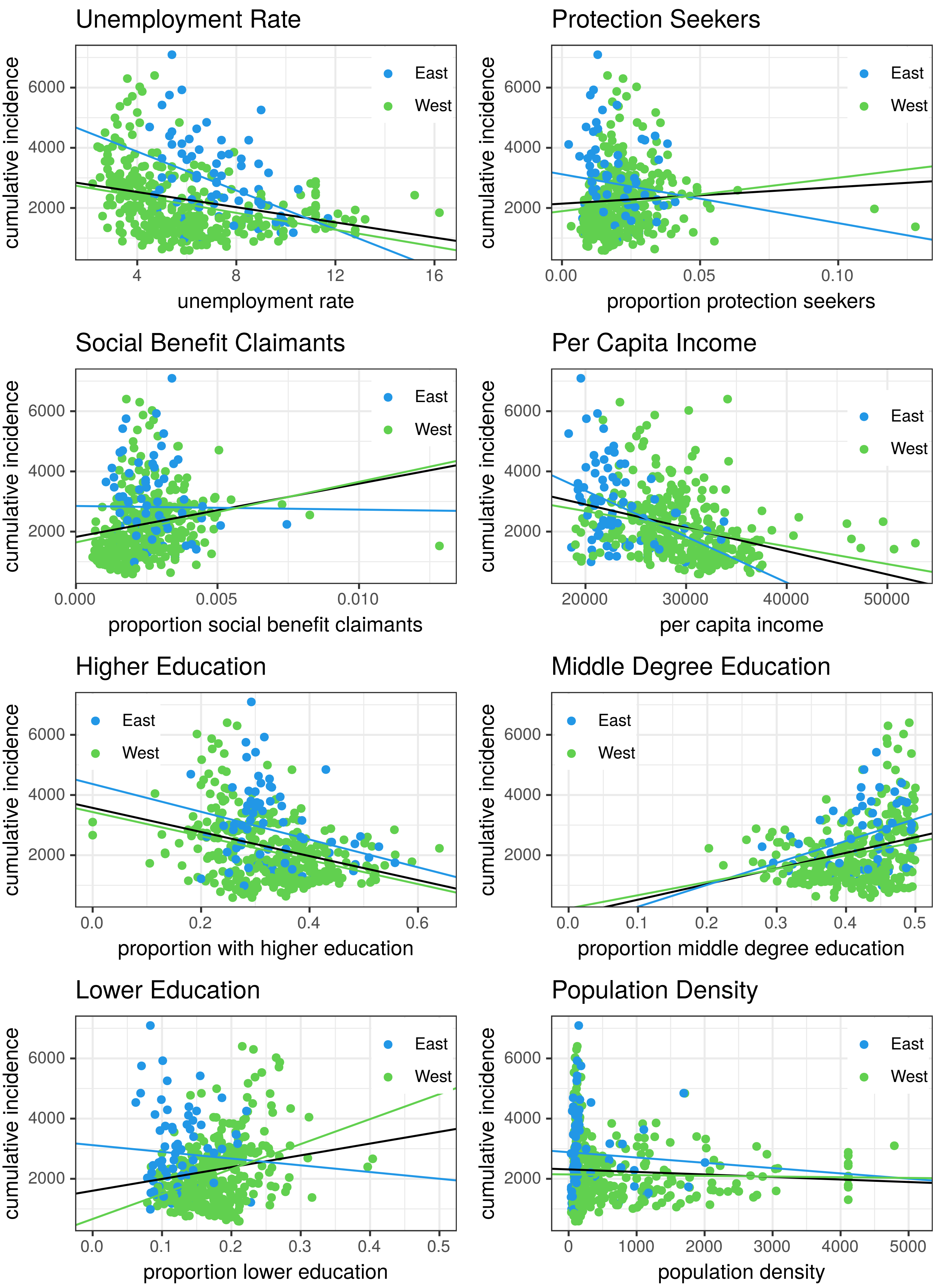

Supplement: Supplementary file 3 [file Data_Sheet_3.PDF]
