# Supplementary material for: Local socio-structural predictors of COVID-19 incidence in Germany
Source: Front Public Health. 2022 Sep 29;10:970092. doi: 10.3389/fpubh.2022.970092 (PMC9556738; doi:10.3389/fpubh.2022.970092)
Supplement: Supplementary file 4 [file Data_Sheet_4.PDF]

Supplement S4 for the article ‘Local Sociostructural Predictors of COVID-19 Incidence in Germany’

Alisha I. Qamar, Leonie Gronwald, Nina Timmesfeld and Hans H. Diebner

2022-04-29

Multicollinearity of the share of votes

The share of votes of all parties add up to 1. Taken as predictors in a regression analysis, this entails some degree of multicollinearity. However, the 411 German rural districts exhibit considerable heterogeneity in terms of popularity of parties expressed by their share of votes. A perfect multicollinearity would be present if and only if the coefficients of collinearity would be identical for all districts. However, the scatterplot shown in Figure-S4-1 below gives an impression of the existing heterogeneity, i.e., the pairwise correlations are moderate such that a regression analysis can still be meaningful.

A common procedure to assess the degree of multicollinearity is to drop one of the variables. We performed a corresponding sensitivity analysis with results shown in Table-S4-1 below. The table depicts the regression results of the full regression model (share of votes of all parties except “other parties” as predictors) and the results of five regression analyses each with one party left out from the set of predictors (except AfD). Leaving one party out leads to an approximately constant shift of the values of the regression parameters of the remaining predictors, whereby the magnitude of the observed shift depends on the omitted party. The observation of such a bias in moderately collinear predictors is a known phenomenon, consequently, as mentioned in the main text, the estimates have to be interpreted in a relative sense. Inferences drawn from these results are unchanged when being compared with the inferences drawn from the full model.

The result of the aforementioned sensitivity analysis can visually quickly be comprehended by throwing a glance onto the forest plot in Figure-S4-2. The relative positions of the CIs within each given model (full model and 5 models with one party dropped) remain approximately invariant.

Figure-S4-1:

Pairwise scatterplots of the share of votes per rural district. The share of votes of all parties add up to 1. Taken as predictors in a regression analysis, this entails multi-collinearity. However, within the given context, pairwise correlations are moderate such that a regression still yields meaningful results (cf. Table-S4-1 below for details). The blue dots correspond to rural districts located in East Germany, whereas the green dots correspond to districts in West Germany.

Pairwise scatterplots of share of votes

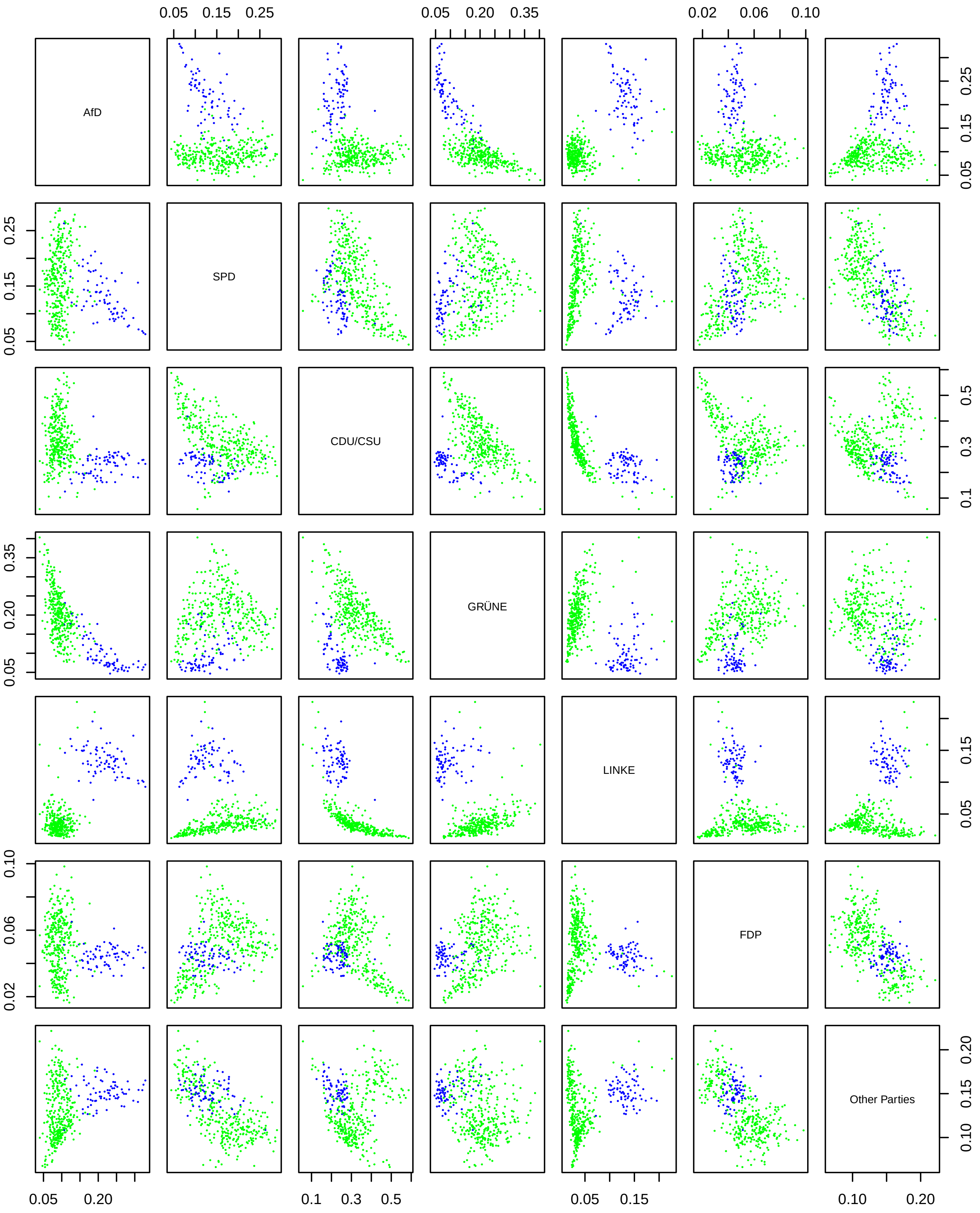

Table-S4-1:

Results of a sensitivity analysis by means of 'leave one party out'. The table depicts the regression results (the β-estimates and p-values) of the full regression model (share of votes of all parties except "other parties" as predictors) and the results of five regression analyses each with one party left out from the set of predictors (except AfD). The denotations of the predictors in the first column are the same as used in Table 2 of the main text and mostly intuitive, whereby the final 6 rows correspond to the party's share of votes. The first two (numeric) columns refer to the full model output. The third and fourth columns belongs to the model with the share of vote of the SPD left out. Since SPD has the strongest negative correlation with AfD, all estimates are shifted by roughly 50 towards higher values. This shift is more or less absorbed by the intercept. All inferences drawn from these results are unchanged when being compared with the inferences drawn from full model. Consequently, as mentioned in the main text, the estimates have to be interpreted in a relative sense. The same holds for all other parties left out as well as can be read from the corresponding columns. Note, p-values with $p = 0.000$ have to be interpreted as $p < 0.0005$.

	Value	p-value	Value	p-value	Value	p-value	Value	p-value	Value	p-value	Value	p-value
(Intercept)	3286.174	0.142	-1592.085	0.268	3523.989	0.014	3856.623	0.016	-2729.525	0.081	1273.098	0.555
Berlin	-75.942	0.767	-300.705	0.221	-66.644	0.787	-60.692	0.810	-543.054	0.017	-349.634	0.148
Brandenburg	350.727	0.220	80.554	0.767	358.989	0.199	370.582	0.187	-246.126	0.304	23.102	0.931
BLBremen	545.600	0.178	70.886	0.849	561.816	0.147	583.093	0.137	-10.454	0.978	168.855	0.664
BW	238.576	0.176	0.961	0.995	247.815	0.128	267.888	0.087	-56.839	0.721	-147.100	0.226
Hamburg	152.558	0.768	-228.148	0.651	166.509	0.742	187.190	0.712	-303.116	0.552	-197.686	0.698
Hessen	18.447	0.930	-316.953	0.068	29.870	0.876	53.511	0.773	-373.992	0.041	-390.522	0.015
MV	-266.537	0.377	-633.003	0.022	-252.740	0.374	-236.840	0.414	-990.465	0.000	-620.130	0.027
Niedersachsen	-167.370	0.417	-608.543	0.000	-151.302	0.374	-124.010	0.460	-619.203	0.000	-491.300	0.006
NRW	318.481	0.120	-74.437	0.623	332.948	0.059	353.890	0.050	-127.585	0.448	-122.419	0.395
RP	218.997	0.261	-93.512	0.564	228.178	0.213	237.091	0.209	-84.885	0.636	-97.865	0.554
SA	365.778	0.241	137.232	0.652	374.763	0.219	385.086	0.210	-301.065	0.244	-37.255	0.896
Saarland	705.270	0.020	291.416	0.274	718.801	0.012	726.343	0.014	222.935	0.420	481.595	0.103
Sachsen	1145.473	0.000	937.941	0.003	1155.134	0.000	1169.463	0.000	581.822	0.041	853.701	0.006
SH	-851.891	0.000	-1218.072	0.000	-837.050	0.000	-802.299	0.000	-1238.142	0.000	-1229.171	0.000
Thüringen	985.378	0.002	693.087	0.019	998.222	0.001	1013.033	0.001	243.242	0.308	631.983	0.029
juveniles	839.001	0.000	839.001	0.000	839.001	0.000	839.001	0.000	839.001	0.000	839.001	0.000
adults	-239.226	0.000	-239.226	0.000	-239.226	0.000	-239.226	0.000	-239.226	0.000	-239.226	0.000
period [61-80]	-374.344	0.000	-374.344	0.000	-374.344	0.000	-374.344	0.000	-374.344	0.000	-374.344	0.000
period [81-100]	1217.538	0.000	1217.538	0.000	1217.538	0.000	1217.538	0.000	1217.538	0.000	1217.538	0.000
lower edu	23.084	0.177	27.185	0.114	23.030	0.178	22.911	0.180	32.530	0.059	26.475	0.125
middle edu	12.294	0.409	15.281	0.308	12.301	0.408	12.305	0.408	20.174	0.178	13.494	0.369
higher edu	15.196	0.298	17.425	0.237	15.221	0.297	15.492	0.288	21.822	0.139	15.562	0.292
density	0.312	0.000	0.323	0.000	0.311	0.000	0.314	0.000	0.309	0.000	0.300	0.000
vote participation	-39.023	0.000	-35.344	0.000	-39.154	0.000	-38.944	0.000	-34.803	0.000	-40.971	0.000
AfD	101.449	0.000	159.824	0.000	98.328	0.000	92.869	0.000	166.607	0.000	122.640	0.000
SPD	-58.548	0.005	NA	NA	-61.136	0.000	-64.769	0.000	-0.452	0.974	-36.541	0.063
CDU	2.694	0.890	52.317	0.000	NA	NA	-3.697	0.657	60.176	0.000	22.433	0.226
GRÜNE	8.521	0.717	63.367	0.000	5.583	0.578	NA	NA	68.338	0.000	29.344	0.196
LINKE	-107.939	0.000	-45.385	0.019	-111.167	0.000	-115.245	0.000	NA	NA	-68.011	0.010
FDP	-100.145	0.003	-66.730	0.035	-101.708	0.001	-103.731	0.001	-43.840	0.148	NA	NA

Figure-S4-2:

Forest plot showing the 95% confidence intervals (CIs) for the estimates corresponding to the party's share of votes. The upper panel contains CIs of the full model (all parties except 'Other parties'). The remaining panels contain CIs from models where one party has been dropped from the list of predictors ('leave one party out'). Obviously, the relative positions of the CIs within each given model remain approximately invariant.

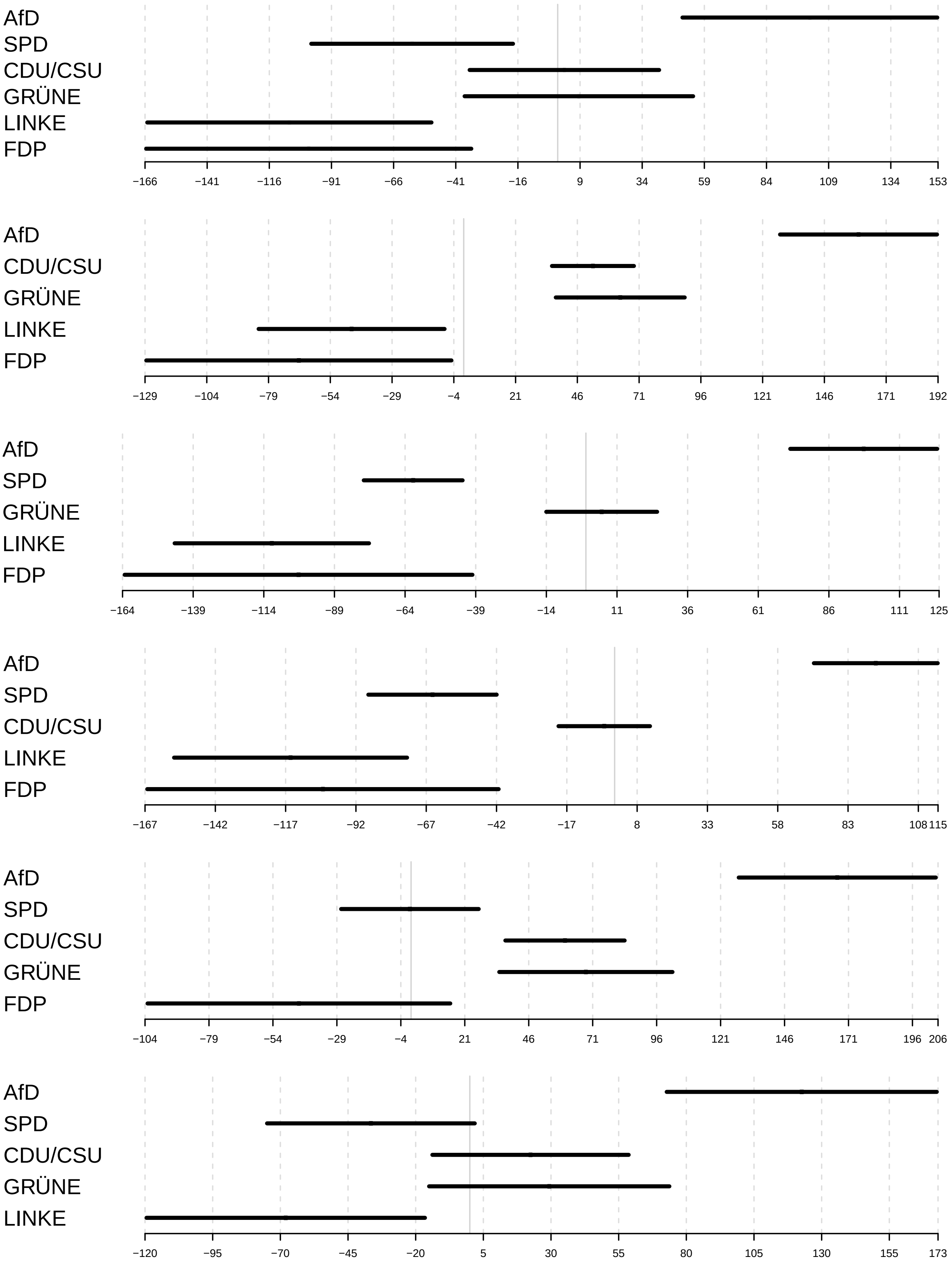
AfD
SPD
CDU/CSU
GRÜNE
LINKE
FDP
−166 −141 −116 −91 −66 −41 −16 9 34 59 84 109 134 153
AfD
CDU/CSU
GRÜNE
LINKE
FDP
−129 −104 −79 −54 −29 −4 21 46 71 96 121 146 171 192
AfD
SPD
GRÜNE
LINKE
FDP
−164 −139 −114 −89 −64 −39 −14 11 36 61 86 111 125
AfD
SPD
CDU/CSU
LINKE
FDP
−167 −142 −117 −92 −67 −42 −17 8 33 58 83 108 115
AfD
SPD
CDU/CSU
GRÜNE
FDP
−104 −79 −54 −29 −4 21 46 71 96 121 146 171 196 206
AfD
SPD
CDU/CSU
GRÜNE
LINKE
−120 −95 −70 −45 −20 5 30 55 80 105 130 155 173
